# Loss of Ufl1/Ufbp1 in hepatocytes promotes liver pathological damage and carcinogenesis through activating mTOR signaling

**DOI:** 10.1186/s13046-023-02681-6

**Published:** 2023-05-03

**Authors:** Fanghui Chen, Le Sheng, Tianci Zhou, Li Yan, Reid Loveless, Honglin Li, Yong Teng, Yafei Cai

**Affiliations:** 1 College of Animal Science and Technology, Nanjing Agricultural University, Nanjing, 210095 China; 2 Department of Hematology and Medical Oncology, Winship Cancer Institute, Emory University School of Medicine, Atlanta, GA 30322 USA; 3 Department of Radiation Oncology, Linyi People Hospital, Linyi, 276000 China; 4 Department of Oral Biology and Diagnostic Sciences, Georgia Cancer Center, Augusta University, Augusta, GA 30912 USA; 5 Department of Biochemistry and Molecular Biology, Medical College of Georgia, Augusta University, Augusta, GA 30912 USA; 6 Wallace H. Coulter Department of Biomedical Engineering, Georgia Institute of Technology & Emory University, Atlanta, GA 30322 USA

**Keywords:** Ufl1/Ufbp1, mTOR, Hepatic fibrosis, Fatty liver, HCC

## Abstract

**Background:**

Ufm1-specific ligase 1 (Ufl1) and Ufm1-binding protein 1 (Ufbp1), as putative targets of ubiquitin-fold modifier 1 (Ufm1), have been implicated in several pathogenesis-related signaling pathways. However, little is known about their functional roles in liver disease.

**Methods:**

Hepatocyte-specific *Ufl1*^Δ/Δhep^ and *Ufbp1*^Δ/Δhep^ mice were used to study their role in liver injury. Fatty liver disease and liver cancer were induced by high-fat diet (HFD) and diethylnitrosamine (DEN) administration, respectively. iTRAQ analysis was employed to screen for downstream targets affected by Ufbp1 deletion. Co-immunoprecipitation was used to determine the interactions between the Ufl1/Ufbp1 complex and the mTOR/GβL complex.

**Results:**

*Ufl1*^Δ/Δhep^ or *Ufbp1*^Δ/Δhep^ mice exhibited hepatocyte apoptosis and mild steatosis at 2 months of age and hepatocellular ballooning, extensive fibrosis, and steatohepatitis at 6–8 months of age. More than 50% of *Ufl1*^Δ/Δhep^ and *Ufbp1*^Δ/Δhep^ mice developed spontaneous hepatocellular carcinoma (HCC) by 14 months of age. Moreover, *Ufl1*^Δ/Δhep^ and *Ufbp1*^Δ/Δhep^ mice were more susceptible to HFD-induced fatty liver and DEN-induced HCC. Mechanistically, the Ufl1/Ufbp1 complex directly interacts with the mTOR/GβL complex and attenuates mTORC1 activity. Ablation of *Ufl1* or *Ufbp1* in hepatocytes dissociates them from the mTOR/GβL complex and activates oncogenic mTOR signaling to drive HCC development.

**Conclusions:**

These findings reveal the potential role of Ufl1 and Ufbp1 as gatekeepers to prevent liver fibrosis and subsequent steatohepatitis and HCC development by inhibiting the mTOR pathway.

**Supplementary Information:**

The online version contains supplementary material available at 10.1186/s13046-023-02681-6.

## Background

Liver fibrosis is the excessive accumulation of extracellular matrix (ECM) proteins (primarily collagen I and collagen III) resulting from wound healing in response to chronic insults such as chronic viral hepatitis, chronic alcohol use, non-alcoholic fatty liver disease (NAFLD), genetic defects, and congenital metabolic disorders. If left untreated, fibrosis can lead to disruption of liver architecture and function over time, resulting in end-stage liver disease (cirrhosis) and increased risk of hepatocellular carcinoma (HCC) [1–4]. Despite its serious impact on global morbidity and mortality, our understanding of the pathogenesis of liver fibrosis continues to evolve. There is increasing evidence that the process can be reversed, but the only effective treatment option is currently transplantation, which is severely limited by organ shortage and graft rejection [5, 6]. Fortunately, many scientists have developed reliable animal models to study ECM modification, turnover and degradation, which may help us identify novel molecular targets that can be used to develop antifibrotic therapies.

A major contributor to fibrosis and liver disease is endoplasmic reticulum (ER) stress, which can be induced by viral infection, NAFLD, or excessive fat accumulation. Upon sensing ER stress, the unfolded protein response (UPR) is activated to restore ER homeostasis by blocking protein translation, increasing the degradation of misfolded proteins, and increasing the production of chaperone proteins. Although the UPR is critical for maintaining cellular homeostasis, it can also promote liver pathogenesis through various pathways, such as those leading to hepatocyte apoptosis and hepatic stellate cell (HSC) activation [7, 8].

Ubiquitin-fold modifier 1 (Ufm1) is a ubiquitin-like protein that has been implicated in the modulation of ER stress and function [9–11]. Beyond its structural similarity to ubiquitin, Ufm1 functions as a post-translational modifier and conjugates to its target proteins in a multi-step process called UFMylation. This process is catalyzed by E1-like ubiquitin-like modifier-activating enzyme 5 (Uba5), E2-like ubiquitin-fold modifier conjugating enzyme 1 (Ufc1), and poorly described E3-Ufm1 ligase components, namely Ufm1-specific ligase 1 (Ufl1, also known as KIAA0776, RCAD, NLBP, and Maxer) in complex with Ufm1-binding protein 1 (Ufbp1, also called DDRGK1, C20orf116, and Dashurin) [12, 13]. Depletion of either *Ufl1* or *Ufbp1* increases ER stress, UPR activation, and apoptosis in mouse bone marrow cells [14]. In addition, Ufbp1 has been found to regulate the NF-κB pathway, and closely interact with the CDK5 regulatory subunit-associated protein 3 (CDK5RAP3, also known as C53 or LZAP), which modulates UFMylation and UPR activation [13, 15, 16]. Meanwhile, Ufbp1 and Ufl1 are critical for starvation-induced ER-phagy [17]. Furthermore, UFMylation dysfunction has been implicated in the pathogenesis of a variety of human diseases, including cancer [18], neuronal diseases [19, 20], heart failure [21], spondyloepiphyseal dysplasia [22, 23], developmental defects [14, 16, 24, 25], gut inflammation [15, 26], and blood disorders [27, 28].

The mammalian target of rapamycin (mTOR), a highly conserved member of the PI3K-related kinases (PIKK) family, is another important constituent in ER stress and liver homeostasis. The primary structure of mTOR consists of five serine/threonine-protein kinase functional and regulatory domains, including N-terminal HEAT repeats and C-terminal KIN, FRB, FAT, and NRD domains [29]. mTOR is divided into two structurally unique complexes, mTOR complex 1 (mTORC1) and mTOR complex 2 (mTORC2), which localize to distinct cellular compartments. mTORC1 is sensitive to rapamycin and can be regulated by growth factors, energy, oxygen, and amino acids; it is also known to accelerate protein synthesis and lipogenesis through the phosphorylation of its substrates, including P70S6, 4EBP-1, and SREBP. On the other hand, mTORC2 responds to growth factors and controls cell growth, proliferation, survival, and migration through its substrates PKC, AKT, and SGK1. In addition, mTORC2 plays a key role in maintaining lipid and glucose balance [30–32].

Recently, we and others have reported that Uba5, Ufl1, and Ufbp1 are involved in mouse development and hematopoiesis. *Ufl1*^−/−^ (E10.5) and *Ufbp1*^−/−^ (E11.5) embryos exhibited small and disorganized fetal livers with decreased cellularity and increased cell death [14]. Furthermore, Ufbp1 deficiency significantly blocked the differentiation of TER119 low cells into TER119 med/high cells, suggesting an indispensable role in both primitive and definitive erythropoiesis during liver development [14]. CDK5RAP3 has been confirmed to be essential for epiboly and gastrulation in zebrafish and to cause prenatal lethality with severe liver hypoplasia when knocked out in mice [25].

Taken together, we and others have subsequently suggested that the Ufm1 conjugation system plays a key role in maintaining ER homeostasis and regulating liver and erythroid development. Here, we investigated the roles of Ufl1 and Ufbp1 in the liver by generating hepatocyte specific *Ufl1*^Δ/Δhep^ and *Ufbp1*^Δ/Δhep^ knockout (KO) mice that exhibited liver fibrosis at 2 months old. Induction of a high-fat diet (HFD) further exacerbates hepatocyte injury and the liver fibrosis phenotype. iTRAQ screening for candidate pathways and signals in *Ufbp1* deficient mouse embryonic fibroblasts (MEFs) revealed the activation of the mTOR signaling pathway. The susceptibility of *Ufl1*^Δ/Δhep^ and *Ufbp1*^Δ/Δhep^ mice to diethylnitrosamine (DEN)-induced carcinogenic liver injury was also found to be increased compared with controls. Finally, the mechanism of Ufl1/Ufbp1-mediated liver fibrosis regulation was investigated and the interaction between the Ufl1/Ufbp1 and mTOR/GβL complexes was confirmed.

## Materials and methods

### Animal models

Rosa 26-Cre/ERT2 *Ufl1*^f/f^ and *Ufbp1*^f/f^ mice (C57BL/6 J) were originally obtained from the laboratory of Dr. Honglin Li (Augusta University), while albumin (Alb) cyclization recombination (Cre) hepatocyte-specific conditional KO mice were obtained from Jackson Laboratory. *Ufl1*^f/f^ or *Ufbp1*^f/f^ mice were crossed with Alb-Cre^+^ mice to generate *Ufl1*^f/f^ and *Ufbp1*^f/f^:Alb-Cre transgenic mice, respectively. After self-crossing the F1 offsprings, we generated hepatocyte-specific KO mice (genotype: *Ufl1*^Δ/Δhep^:Alb-Cre and *Ufbp1*^Δ/Δhep^:Alb-Cre). Cre recombinase was used on the 7^th^ exon of the *Ufl1* gene and the 3^rd^ and 4^th^ exons of the *Ufbp1* gene. Genotyping primers: Albcre F-ACCTGAAGATGTTCGCGATTATCT; AlbCre R-ACCGTCAGTACGTGAGATATCTT. To obtain Ufbp1 KO mouse embryos, the following primers were used for PCR genotyping: P1-TAGTACTTGAAGTCTGGCTTGGTA, P2-CACAACGGGTTCTTCTGTTAGTCC, and P3-TAGTCAGGAACTGATGAGTGTCTC. Mice were maintained in filter-topped cages and were given free access to autoclaved regular normal diet or HFD (added additional 12% fat from lard, 2% cholesterol based on normal diet-fed mice content) and water. Male mice of indicated age were used for the fatty liver model. HCC was induced chemically induced by intraperitoneal injection of 12 mg/kg DEN (Sigma-Aldrich, St Louis, MO) in 8-week-old male *Ufl1* and *Ufbp1*^Δ/Δhep^: Alb-Cre (hepatocyte specific) mice. Six months after DEN injection, the chemically induced HCC was evaluated. Rapamycin (MedChemExpress, Monmouth Junction, NJ) was dissolved in 100% dimethyl sulfoxide (DMSO) to prepare a stock solution of 20 mg/ml. It was diluted with phosphate buffered saline (PBS) in vitro and corn oil in vivo as working solution. All animal experiments were approved by the Animal Ethics Committee of Nanjing Agricultural University (31,672,512).

### Cell lines and isolation and immortalization of MEFs

Human HCC HepG2, and Hep3B cell lines were obtained from ATCC (Manassas, Virginia). All cell lines were maintained in the growth medium according to the manufacturer's instructions and passages less than 5 were used in this study. To generate MEFs, pregnant mice were sacrificed for 14-day embryos. The head and red organs were removed and thoroughly minced until the tissue could be pipetted. 1 mL of 0.05% trypsin/EDTA (Gibco, Invitrogen) containing 100 Kunitz units of DNase I (Invitrogen) was added per embryo. The mixture was incubated at 37 °C for 20 min, and the cells were dissociated by repeated up and down pipetting at 5-min intervals. Approximately 1.5 volume of fresh DMEM medium (containing 10% FBS, 1/100 L-glutamine (200 mM) and penicillin–streptomycin, v/v) was then added to ensure trypsin inactivation. The cell pellet was collected in warm complete medium at low speed (350 × g) for 8 min. A large number of cells was equivalent to 2–3 embryos in each T25 flask coated with 0.2% gelatin (bovine dermal gelatin, type B, Sigma) for 2 h. Passage 0 (P0) fibroblast cells reaching 80%-90% confluence were passaged and cultured until P3, then LT lentivirus was used for stable transfection and positive immortalized cells were screened with puromycin.

### iTRAQ assay

iTRAQ assay was performed by Wuhan Jin Kairui Biological Engineering Company (Wuhan, China). Briefly, 4-OHT and EtOH samples were labeled with the iTRAQ Reagent-8 plex Multiplex Kit (A B Sciex UK Ltd). The labeled samples were fractionated on a high-performance liquid chromatography (HPLC) system (Thermo DINOEX Ultimate 3000 BioRS) using a Durashell C18(5 um, 100 Å, 4.6 × 250 mm). LC–ESI–MS/MS analysis was performed on an AB SCIEX nanoLC-MS/MS (Triple TOF 5600 plus) system. The original MS/MS file data were submitted to ProteinPilot v4.5 software for data analysis. To ensure the reliability and significant changes of the data, 1.5-fold change and P ≤ 0.05 were set as the threshold compared to the control (EtOH group). Genesis software was used for hierarchical clustering applied to differentially expressed proteins. All identified sequences were first searched for homology using a localized NCBI blast program against the NCBI animal database. Gene Ontology terms (http://geneontology.org/) were used to map the biological and functional properties of all identified proteins. GO term matching was performed using the blast2go v4.5 pipeline.

### Blood sample measurement

Mouse blood samples were immediately centrifuged at 2,500 × g for 10 min at 4 °C to isolate serum. Aspartate aminotransferase (AST), alanine aminotransferase (ALT), and triglycerides (TG) levels were determined using an autoanalyzer (7020 Automatic Analyzer, Hitachi, Tokyo, Japan) according to the manufacturer's instructions.

### Apoptosis

The Annexin-V-FITC apoptosis detection (Invitrogen, USA) of Primary Mouse Hepatocyte was performed using FACS caliber Flow cytometer (BD Biosciences, USA) and Flow Jo 10.0 software (Tree Star, San Carlos, CA). The in situ cell death detection kit (TMR Red, Roche, Basel, Switzerland) was used to determine the cell apoptosis ratio according to the manufacturer’s instructions.

### Liver histological analysis

Fresh liver tissues were rapidly fixed in 4% paraformaldehyde overnight at room temperature and embedded in paraffin. The 4-μm thick liver tissue sections were processed for H&E, Oil Red O (Sigma), Masson's and Sirius Red (Sigma) staining. Sections were deparaffinized and rehydrated according to the manufacturer's instructions. Positive areas were quantified using Image J software (NIH).

### Transmission electron microscopy (TEM)

Briefly, 1 mm^3^ of liver tissues were fixed with 2.5% glutaraldehyde in 0.1 M phosphate buffer (pH 7.4). After fixation, cells were stained with osmium tetroxide, dehydrated, and embedded in Epon-Araldite resin. Ultrathin sections were stained with uranyl acetate and lead citrate and observed by TEM (HT7650, Hitachi).

### Co-immunoprecipitation (IP)

Cells from fresh mouse liver tissues were lysed with Pierce™ IP Lysis Buffer (Thermo Fisher Scientific, Waltham, MA), with the addition of Protease Inhibitor Cocktail Tablets (Roche, Basel, Switzerland). Lysate (1500 μg total protein) was incubated with the antibodies against Ufl1, Ufbp1, mTOR, GβL or IgG, followed by addition of protein A/G plus agarose beads (Santa Cruz Biotechnology, Dallas, TX) overnight at 4 °C on a rotating platform. Beads were washed six times in lysis buffer, and proteins were eluted with 2 × Laemmli buffer (100 mm Tris–HCl, pH 6.8, 20% glycerol, 4% SDS, 10% β-mercaptoethanol) and heated to 95 °C for 5 min before western blot. Anti-rat Ufl1 and Ufbp1 polyclonal antibodies used in this study were generated in our laboratory. All affinity-purified and species-specific HRP- and fluorophore-conjugated secondary antibodies were obtained from Zhong Shan Jin Qiao (Beijing, China) or Cell Signaling Technology (Danvers, MA). The commercial antibodies are listed in Supplementary Table S1.

### Quantitative RT-PCR (QRT-PCR)

Total RNA was isolated using Gene JET RNA Purification Kit (Thermo Fisher Scientific) or Trizol reagent (Invitrogen), and then reverse transcribed using AMV reverse transcriptase and random primers according to the manufacturer's instructions (Thermo Fisher Scientific). iTaq Universal SYBR Green Supermix Kit (BIO-RAD, Hercules, CA), the running program: 40 cycles of 95 °C for 15 s and 60 °C for 1 min on ABI 7500 Real-Time PCR System (Thermo Fisher Scientific). The results were analyzed by QuantStudioTM 7 Flex system software using the 2^ (-delta delta Ct) method. Genes were normalized to endogenous GAPDH or β-actin. Primers are listed in Supplementary Table S2.

### Tissue microarray and immunohistochemistry (IHC)

Tissue arrays of human liver cancer samples were purchased from Shanghai Outdo Biotech Company (Cat No. HlivH150CS05; Lot No. XT19-014), including 75 pairs of HCC tissues and matched normal tissues with complete clinicopathologic information. Immunohistochemical staining for Ufl1, Ufbp1, α-SMA, p-mTOR, Ki67, and AFP was performed using the SP Rabbit & Mouse HRP Kit (CW2069; CWBIO). HRP activity was determined by diaminobenzidine (DAB) staining.

### Immunofluorescent staining

Briefly, liver tissues were fixed with 4% formaldehyde in PBS for 15 min and permeabilized with 0.1% Triton X-100 in PBS for 10 min. After blocking for 1 h, liver sections were incubated with primary antibodies overnight at 4 °C, followed by incubation for 1 h with Alexa Flour 488-conjugated donkey anti-rabbit IgG (Abcam, Cambridge, United Kingdom) or Alexa Flour 555-conjugated donkey anti-mouse IgG (Abcam). Representative fluorescence images were captured with a Carl-Zeiss 900 laser scanning microscope and analyzed using Zeiss 3.0 software.

### Statistical analysis

Statistical significance was assessed by two-tailed unpaired nonparametric or Student’s t-test using Prism 8 software (GraphPad, CA). Statistical differences in tumor progression between two groups of experimental mice were analyzed by multiple t-tests at each tumor measurement time point using Prism 8. The association between IHC score and Ufl1/Ufbp1 expression was analyzed by chi-squared test. All data were presented as mean ± standard deviations (SDs). Differences were considered statistically significant when *p* < 0.05.

## Results

### Hepatocyte-specific deletion of *Ufl1* or *Ufbp1* in mice results in liver injury

**Fig. 1:**
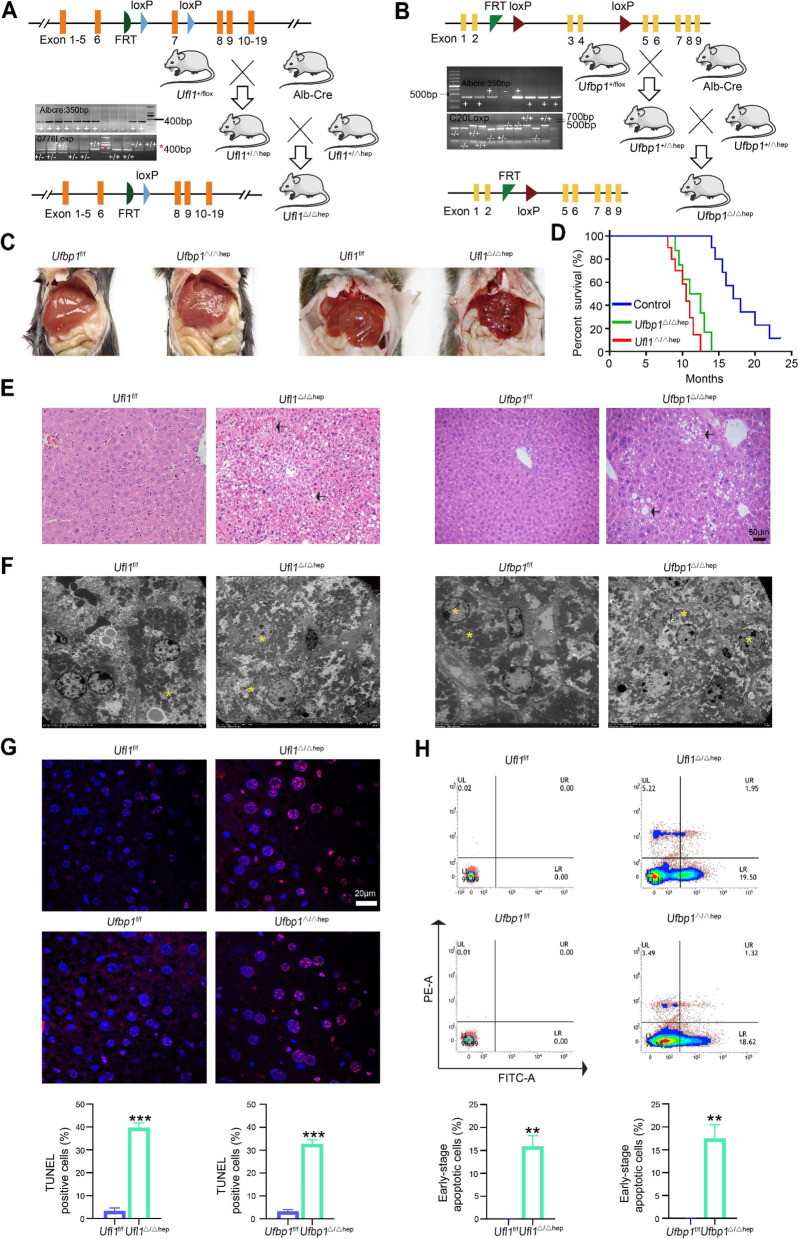
Hepatocyte-specific deletion of *Ufl1* or *Ufbp1* in mice results in liver injury. **A** *Ufl1* floxed allele and generation of hepatic *Ufl1* knockout mice. **B** *Ufbp1* floxed allele and generation of hepatic *Ufbp1* knockout mice. **C** Representative gross anatomy of liver tissues extracted from 2-month-old *Ufl1*^Δ/Δhep^, *Ufbp1*^Δ/Δhep^, and control mice. **D** Survival curve of *Ufl1*^Δ/Δhep^, *Ufbp1*^Δ/Δhep^, and control mice (*n* = 30 mice/group). **E** Histochemical analysis of liver tissues extracted from 2-month-old *Ufl1*^Δ/Δhep^, *Ufbp1*^Δ/Δhep^, and control mice by HE analysis. Arrows indicate injury positions. **F** Representative TEM image of liver tissues extracted from 2-month-old *Ufl1*^Δ/Δhep^, *Ufbp1*^Δ/Δhep^, and control mice. Stars indicate apoptotic cells. **G** Apoptosis analysis of liver tissues extracted from 2-month-old *Ufl1*^Δ/Δhep^, *Ufbp1*.^Δ/Δhep^, and control mice by TUNEL staining. TUNEL-positive cells were quantified (*n* = 5 mice/group) and are shown in the lower panel. **H** Apoptosis of primary mouse hepatocytes measured by flow analysis. Representative results and quantitative data (*n* = 5 mice/group) are shown in the upper and lower panels, respectively. ** *p* < 0.01

As critical components of the Ufm1 conjugation system, depletion of Ufl1 or Ufbp1 has been reported to lead to extensive cell death in the liver of *Ufl1*^*−/−*^ or *Ufbp1*^−/−^ embryos [14]. Here, we found that the absence of *Ufl1* or *Ufbp1* in hepatocytes caused phenotypic changes, which was consistent with previous reports. To generate hepatocyte-specific *Ufl1* KO mice, *Ufl1*^+/f^ mice were mated with Alb-Cre mice, and *Ufbp1*^Δ/Δhep^ mice were generated by a similar mating pattern. Briefly, *Ufl1* exon 7 was flanked by loxP sites, and *Ufbp1* was followed by exons 3 and 4. Insertion of the gene trap was confirmed by genomic PCR (Fig. 1A-B) and KO efficiency as evidenced by Western blot (Supplementary Fig. S1A). At 2 months of age, livers were harvested from KO mice and showed hepatic pathological changes and localized lesions that became rougher and more irregular compared to the smooth surface of littermate control livers. Although both KO models exhibited this phenotype, it was more severe in *Ufl1*^Δ/Δhep^ mice than *Ufbp1*^Δ/Δhep^ mice (Fig. 1C). The percentage of the liver to body weight, however, was found to significantly increase from 4% to 5.4% in *Ufbp1*^Δ/Δhep^ mice (Supplementary Fig. S1B). Based on the survival curve analysis, both KO mouse models died between 10–14 months (Fig. 1D). These results suggest an essential role of *Ufl1* and *Ufbp1* in prolonging hepatocyte survival.

It is worth noting that both KO livers appeared to have relatively intact hepatic lobular architecture but obscured intercellular borders and mild hepatocyte steatosis. Specifically, in addition to the formation of collagen-like fibrosis around the central veins, nuclear proliferation and vacuole formation in the cytoplasm were observed after *Ufl1* ablation. *Ufbp1*^Δ/Δhep^ mice appeared to have obvious vacuolar degeneration of varying sizes in hepatocytes (Fig. 1E). TEM observation also revealed that mitochondria with normal cristae accumulated around the nuclei of WT hepatocytes, whereas mitochondria in both *Ufl1*^Δ/Δhep^ and *Ufbp1*^Δ/Δhep^ hepatocytes showed abnormal cristae and swelling. In addition, the nucleus in KO hepatocytes showed shrinkage and deformation, while the cytoplasm was loosely arranged (Fig. 1F). Apoptotic cells in liver tissues were examined by TUNEL staining, and the percentage of TUNEL-positive cells was 30–40% higher in *Ufl1*^Δ/Δhep^ and *Ufbp1*^Δ/Δhep^ mice than in their respective controls (Fig. 1G). The percentage of apoptotic primary hepatocytes isolated from the livers was further determined by flow analysis using Annexin V-FITC/PI staining, which showed that approximately 20% of KO hepatocytes were in the early stage of apoptosis compared to a negligible percentage of control hepatocytes (Fig. 1H). Taken together, these results strongly suggest that specific ablation of Ufl1 or Ufbp1 in hepatocytes results in severe liver injury.

### Hepatic *Ufl1* or *Ufbp1* deficiency increases the susceptibility to HFD-induced fatty liver

**Fig. 2:**
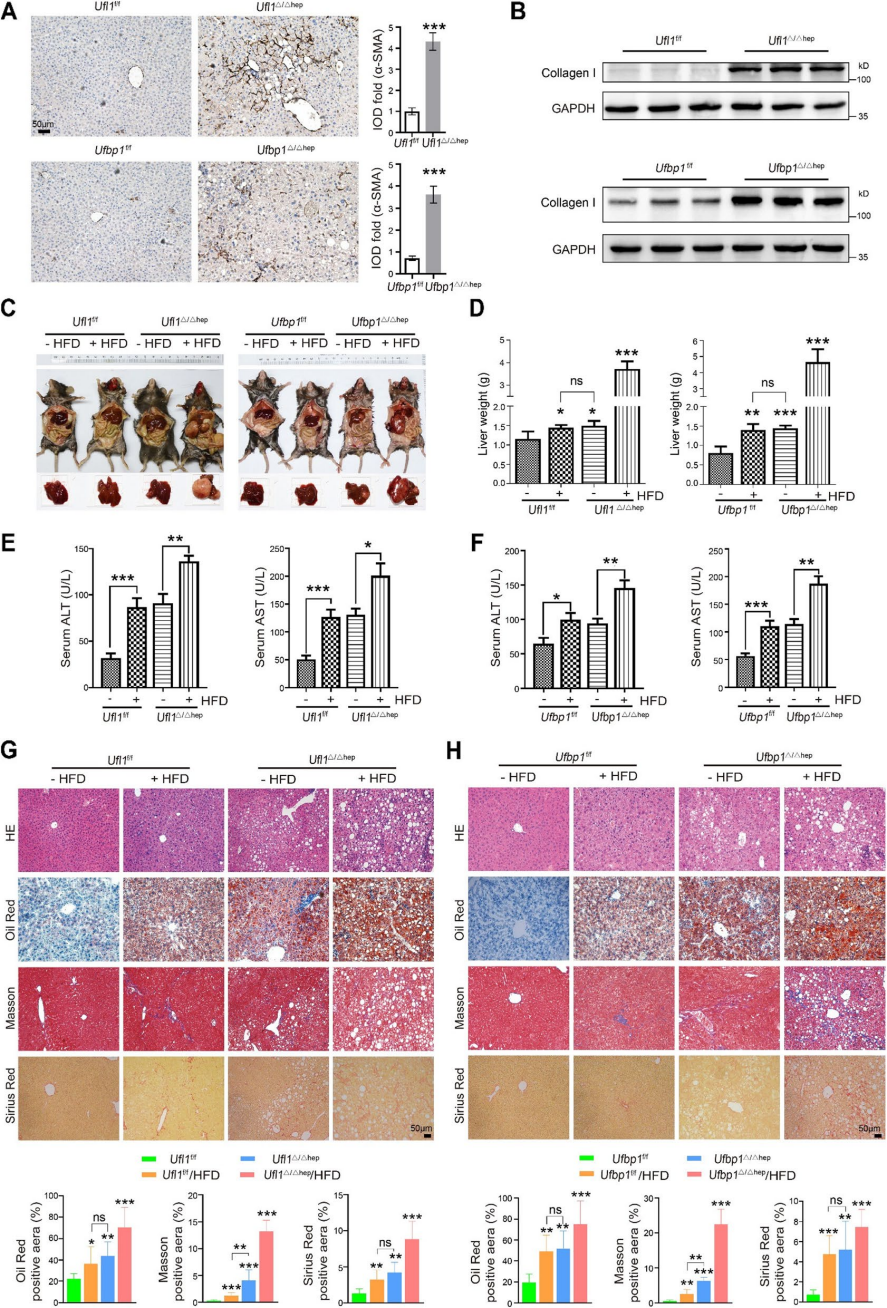
Hepatic *Ufl1* or *Ufbp1* deficiency increases the susceptibility to HFD-induced fatty liver. **A** Immunostaining of α-SMA in liver tissues extracted from *Ufl1*^Δ/Δhep^, *Ufbp1*^Δ/Δhep^, and control mice. Quantitative data are shown in the right panel (*n* = 5 mice/group). **B** Western blot analysis of collagen I protein levels in liver tissues extracted from *Ufl1*^Δ/Δhep^, *Ufbp1*^Δ/Δhep^, and control mice. **C** Anatomic illustration of liver tissues extracted from *Ufl1*^Δ/Δhep^, *Ufbp1*^Δ/Δhep^, mice and control mice after 12 weeks of HFD feeding. **D** Changes in liver weight in *Ufl1*^Δ/Δhep^, *Ufbp1*^Δ/Δhep^, and control mice after 12 weeks of HFD feeding (*n* = 5 mice/group). **E**, **F** Assessment of liver injury in *Ufl1*^Δ/Δhep^, *Ufbp1*^Δ/Δhep^, and control mice through serum ALT and AST levels (*n* = 5 mice/group). **G**, **H** Detection of liver injury and fibrosis levels in *Ufl1*^Δ/Δhep^, *Ufbp1*.^Δ/Δhep^, and control mice using four different staining approaches. HE, Masson, and Sirius Red staining were used to determine the extent of liver fibrosis, and Oil Red O staining was used to detect adipocyte lipid droplets. Quantitative data are shown in the lower panels (*n* = 5 mice/group). * *p* < 0.05; ** *p* < 0.01; *** *p* < 0.001

To determine whether Ufl1 or Ufbp1 depletion-mediated liver injury is associated with liver disease, we examined the expression of known implicated components in our models. In particular, α-SMA is associated with the activation of HSCs into myofibroblast-like cells and is implicated in liver cirrhosis and cancer. In contrast to KO control livers, α-SMA expression was significantly increased in the livers of 6-month-old *Ufl1*^Δ/Δhep^ and *Ufbp1*^*Δ*/Δhep^ mice (Fig. 2A). Compared with KO control mice, the levels of collagen I protein (a marker of liver fibrosis) were also profoundly elevated, underscored by a 6.43- and 4.15-fold increase in mRNA expression in *Ufl1*^Δ/Δhep^ and *Ufbp1*^*Δ*/Δhep^ mice, respectively (Fig. 2B and Supplementary Fig. S2A). These observations suggest that hepatic Ufl1 or Ufbp1 deficiency increases liver fibrosis.

We next explored the association of hepatic *Ufl1* or *Ufbp1* deficiency with the susceptibility to HFD-induced fatty liver. After 3 months of feeding *Ufl1*^Δ/Δhep^ and *Ufbp1*^Δ/Δhep^ mice a HFD (+ HFD) or standard diet (-HFD), gross anatomy revealed that almost all livers in the + HFD groups were occupied by a marked accumulation of yellow fat, a typical symptom of severe steatosis with early cirrhosis, and further appeared rough and to have multiple white nodule lesions (Fig. 2C). In comparison, KO control mice in the -HFD groups showed no signs of liver tissue damage and, in the + HFD groups, the morphological characteristics of lesions were especially difficult to appreciate through macroscopic observation (Fig. 2C). The liver weights of *Ufl1*^Δ/Δhep^ and *Ufbp1*^Δ/Δhep^ mice in the + HFD groups were also found to be approximately fourfold heavier than normal (Fig. 2D). Moreover, the liver/body weight ratio of *Ufl1*^Δ/Δhep^ and *Ufbp1*^Δ/Δhep^ mice in the + HFD groups remarkably increased to approximately 12%, compared to the ~ 5% ratio seen in KO control mice in the + HFD animals (Supplementary Fig. S2B). The serum concentrations of ALT and AST (markers of liver injury) were also greatly increased in the *Ufl1*^Δ/Δhep^ and *Ufbp1*^Δ/Δhep^ mice compared to KO control mice fed a standard diet, which was to a more extreme under the influence of HFD feeding (Fig. 2E-F).

Through H&E staining, proliferated fatty hepatocytes shaped like vacuoles were seen surrounding central veins in the livers of *Ufl1*^Δ/Δhep^ and *Ufbp1*^Δ/Δhep^ mice (Fig. 2G-H). HFD uptake further accelerated the steatosis phenotype in KO mice, leading to the accumulation of larger and more fatty vacuoles, inflammatory cell infiltration, and disruption of hepatocyte arrangement. In contrast, KO control mice in the + HFD groups presented only slight hepatic steatosis (Fig. 2G-H). Oil Red O staining confirmed that HFD feeding induced greater adipocyte proliferation and fatty liver in *Ufl1*^Δ/Δhep^ and *Ufbp1*^Δ/Δhep^ mice, as seen by larger areas of tightly packed lipid droplets (Fig. 2G-H). Accompanying these fatty hepatocytes were a large number of proliferated fibroblasts and an accumulation of collagenous fiber (blue) deposited in the hepatic lobule of mice following HFD feeding, as seen through Masson’s staining (Fig. 2G-H). This, together with Sirius red staining, revealed a greater ratio of collagenous fiber proliferation and fibrosis in *Ufl1*^Δ/Δhep^ and *Ufbp1*^Δ/Δhep^ mouse livers in the + HFD groups compared with KO control mouse livers (Fig. 2G-H). Collectively, results from these analyses suggest that *Ufl1* or *Ufbp1* deletion increases the susceptibility to HFD-induced fatty liver.

### *Ufl1* or* Ufbp1* ablation activates the mTOR signaling pathway

**Fig. 3:**
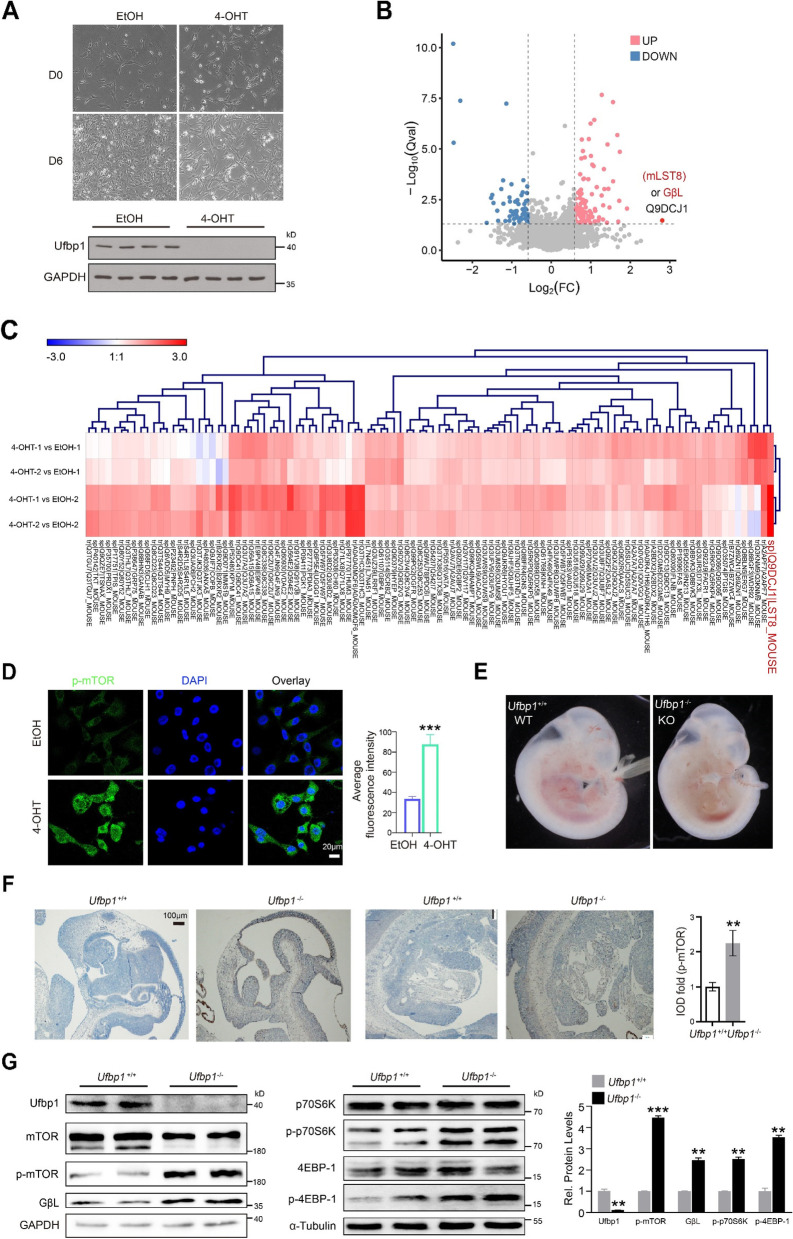
*Ufbp1* deficiency leads to activation of the mTOR signaling pathway in MEFs and mouse E11.5 embryos. A Loss of Ufbp1 expression in *Ufbp1*^f/f^: Rosa26Cre-ERT2 mouse MEFs following 4-OHT treatment (low panel) and the resulting cell morphological changes (upper panel). **B** Volcano plots for the DE proteins in *Ufbp1*^f/f^: Rosa26Cre-ERT2 mouse MEFs treated with 4-OHT (vs EtOH). **C** A heat map of partial DE proteins based on iTRAQ data. Proteins were extracted from E14.5d *Ufbp1*^f/f^: Rosa26Cre-ERT2 mouse MEFs treated with 4-OHT or EtOH for 6 days, followed by iTRAQ analysis. **D** Immunofluorescence of p-mTOR in *Ufbp1*^−/−^ and control mouse MEFs. Quantitative data are shown in the right panel (*n* = 12 random selected fields). **E** E11.5 embryos of *Ufbp1*^−/−^ and control mice. **F** Immunostaining of p-mTOR throughout entire E11.5 embryos of *Ufbp1*^−/−^ and control mice. **G** Western blot analysis of the key molecules involved in the mTOR signaling from samples extracted from E11.5 embryos of *Ufbp1*.^−/−^ and control mice. Quantitative data are shown in the right panel. All western blots were independently repeated at least three times with consistent results. ** *p* < 0.01; *** *p* < 0.001

To investigate how *Ufl1* or *Ufbp1* deletion induced liver damage, we first harvested and immortalized MEFs from an E14.5d mouse embryo (genotype: *Ufbp1*^f/f^: Rosa26Cre-ERT2). Four-hydroxytamoxifen (4-OHT) was then used in the Cre-mediated deletion of *Ufbp1* in MEFs, while ethanol (EtOH) was used as a control. After 6 days of 4-OHT treatment, it was clear that *Ufbp1* had been effectively deleted (Fig. 3A). At this point, it was also evident that the 4-OHT treated MEFs (*Ufbp1*^−/−^) grew much slower than EtOH-treated controls and exhibited morphological changes, namely wrinkled membranes and enlarged intracellular spaces (Fig. 3A). Cell viability of *Ufbp1*^−/−^ MEFs was also found to be roughly half that of EtOH control cells on day 6, as revealed by crystal violet staining (Supplementary Fig. S3A). Using iTRAQ-based proteomic analysis, we then screened for potential downstream targets of Ufl1 and Ufbp1. A total of 106 upregulated and 61 downregulated proteins were identified in this study, and the top 15 most up- and downregulated differentially expressed (DE) proteins are listed in Supplementary Table S3. We found that the mTOR signaling was the most activated pathway upon *Ufbp1* deletion (Fig. 3B-C). Volcano plot and hierarchical clustering analysis were subsequently performed across the treatment and control groups, which showed GβL (also known as mLST8), a component of the mTORC complex, had the highest contribution level (2.8-fold) among all upregulated proteins (Fig. 3B-C). Gene ontology analysis based on upregulated and downregulated DE proteins revealed that cellular process, metabolic process, biological regulation, and regulation of biological process were the four greatest upregulated processes (Supplementary Fig. S3B), all of which involved the mTOR pathway. Through immunofluorescence and quantitation, p-mTOR level was also seen to be significantly increased and localized to the cytoplasm of *Ufbp1*^−/−^ MEFs in comparison with EtOH controls (Fig. 3D).

To further explore the relationship between Ufbp1 and mTOR signaling activation, live whole-body *Ufbp1*^*−/−*^ mouse embryos were harvested at E11.5d (Fig. 3E), given that embryonic lethality was exhibited at E13.5d. Through subsequent IHC, p-mTOR expression was seen to be widely and evenly distributed and significantly higher in KO mouse embryos than KO controls (Fig. 3F). In addition to p-mTOR, the levels of GβL, p-4EBP-1, and p-p70S6 proteins were also markedly increased in KO mouse embryos compared with controls (Fig. 3G).

**Fig. 4:**
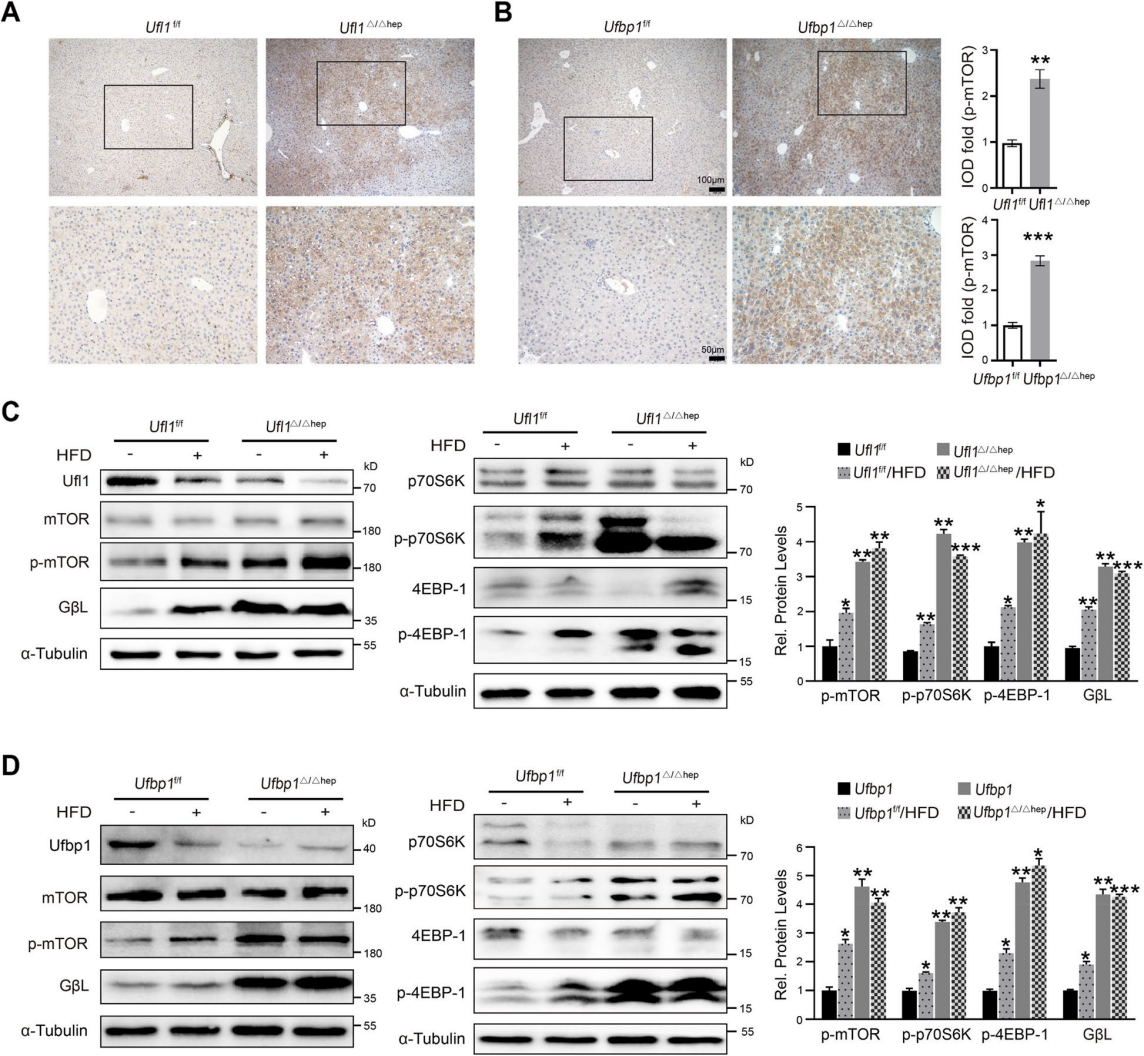
Hepatic *Ufl1* or *Ufbp1* deficiency leads to activation of the mTOR signaling pathway. **A**, **B** Immunostaining of p-mTOR in liver tissues extracted from 6-month-old *Ufbp1*^Δ/Δhep^, *Ufbp1*^Δ/Δhep^, and control mice (*n* = 5 mice/group). **C**, **D** Western blot analysis of the key molecules involved in the mTOR signaling in *Ufl1*^Δ/Δhep^, *Ufbp1*.^Δ/Δhep^, and control mice with or without 12 weeks of HFD feeding. Quantitative data are shown in the right panel. All western blots were independently repeated at least three times with consistent results. ** *p* < 0.01; *** *p* < 0.001

Furthermore, quantification of IHC liver slides from *Ufbp1*^Δ/Δhep^ and *Ufl1*^Δ/Δhep^, compared with control mice reinforced that p-mTOR expression levels were significantly higher in the absence of *Ufbp1* (*p* < 0.001) and *Ufl1*(*p* < 0.01); furthermore, we observed more p-mTOR localized in the cytoplasm (Fig. 4A-B). Using the previous fatty liver model, it was further revealed that HFD induction in KO control mice led to mTOR signaling pathway activation in the liver, as evidenced by increased levels of p-mTOR, GβL, p-p70S6, and p-4EBP-1 (Fig. 4C-D). Although there were only slight differences in mTOR signaling pathway activation in *Ufl1*^Δ/Δhep^ and *Ufbp1*^Δ/Δhep^ mice with or without HFD feeding, compared to KO controls, mTOR signaling was significantly upregulated in the livers of KO mice (Fig. 4C-D). mTOR has emerged as a major regulator of autophagy and, interestingly, we detected the accumulation of LC3-II and upregulation of p62 (SQSTM1) (autophagosome markers) along with genes downstream of mTOR in liver samples from *Ufl1*^Δ/Δhep^ and *Ufbp1*^Δ/Δhep^ mice (Supplementary Fig. S4B). LC3B was significantly increased in the livers of 8-month-old *Ufl1*^Δ/Δhep^ mice (Supplementary Fig. S4C). Immunofluorescence additionally revealed that approximately 12.5% of *Ufbp1*^−/−^ MEFs possessed LC3-positive autophagosomes (Supplementary Fig. S4D). Taken together, these results demonstrate that *Ufl1* or *Ufbp1* ablation activates the mTOR signaling pathway and its downstream signaling molecules.

### Loss of hepatic *Ufl1* or *Ufbp1* induces spontaneous HCC

**Fig. 5:**
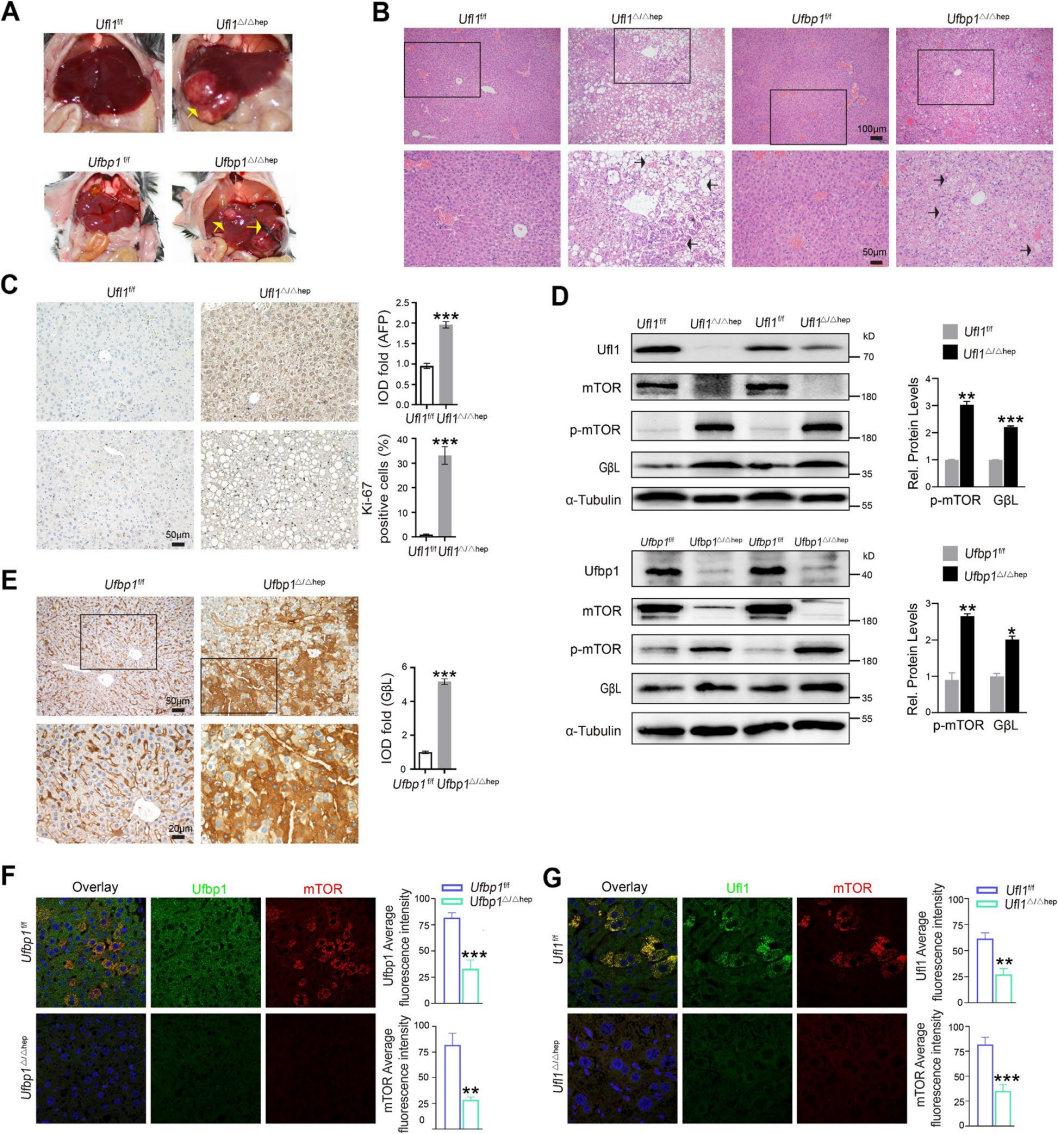
Hepatic *Ufl1* or *Ufbp1* deficiency results in spontaneous liver carcinomas in 14-month old mice. **A** Anatomic illustration of liver tissues extracted from 14-month-old *Ufl1*^Δ/Δhep^, *Ufbp1*^Δ/Δhep^, and control mice. **B** Histological analysis of liver tissues extracted from 14-month-old *Ufl1*^Δ/Δhep^, *Ufbp1*^Δ/Δhep^, and control mice by H&E staining. Arrow: foam cells/macrophages/apoptotic cells. **C** Immunostaining of the liver tumor marker AFP and the proliferation marker Ki67 in liver tissues extracted from 14-month-old *Ufl1*^Δ/Δhep^ and control mice. Quantitative data are shown in the right panel (*n* = 5 mice/group). **D** Western blot analysis of the key molecules involved in mTOR signaling from samples extracted from 14-month-old *Ufl1*^Δ/Δhep^, *Ufbp1*^Δ/Δhep^, and control mice. All western blots were independently repeated at least three times with consistent results. Quantitative data are shown in the right panel. **E** Immunostaining of GβL in liver tissues extracted from 14-month-old *Ufbp1*.^Δ/Δhep^ and control mice. Quantitative data are shown in the right panel (*n* = 5 mice/group). Immunofluorescence showing colocalization of Ufl1 and mTOR **F** or Ufbp1 and mTOR **G** in 14-month-old mice. Quantitative data are shown in the right panel (*n* = 5 mice/group). ** *p* < 0.01; *** *p* < 0.001

By 2 months of age, *Ufl1*^Δ/Δhep^ and *Ufbp1*^Δ/Δhep^ mice exhibited early liver damage and mild steatosis, and by 6–8 months, hepatocellular ballooning, extensive fibrosis, mild steatohepatitis, and infiltration of inflammatory cells. Moreover, over 50% of these KO mice suffered from spontaneous HCC when 14–16 months old (Supplementary Fig. S5A), which presented as white nodules of varying sizes (Fig. 5A). Liver tissues from 14-month-old KO mice were also revealed to have deformed hepatic lobules and nuclei and to exhibit foam cells seemingly ubiquitously, suggestive of HCC (Fig. 5B). Compared to *Ufl1*^f/f^ mice, higher levels of the tumor marker alpha-fetoprotein (AFP) were also seen in *Ufl1*^Δ/Δhep^ livers, as well as a ~ 30% increase in Ki67-positive cells, which is strongly indicative of rapid tumor growth (Fig. 5C). Similarly, relative to *Ufbp1*^f/f^ mice, the mRNA expression of AFP in *Ufbp1*^Δ/Δhep^ mice was found to be eightfold and 30-fold greater at 2 months and 14 months of age, respectively (Supplementary Fig. S5B). During liver carcinogenesis, hepatic depletion of *Ufl1* or *Ufbp1* in 14-month-old mice was accompanied by a decrease in mTOR levels but juxtaposed by increased levels of p-mTOR and GβL (Fig. 5D). In normal 14-month-old mouse liver tissue, both Ufl1 and Ufbp1 colocalized with mTOR in the cytoplasm, and ablation of either significantly reduced total mTOR levels (Fig. 5F-G). Additionally, the increased staining intensity for GβL was observed in the cytoplasm of *Ufbp1*^Δ/Δhep^ livers (Fig. 5E).

**Fig. 6:**
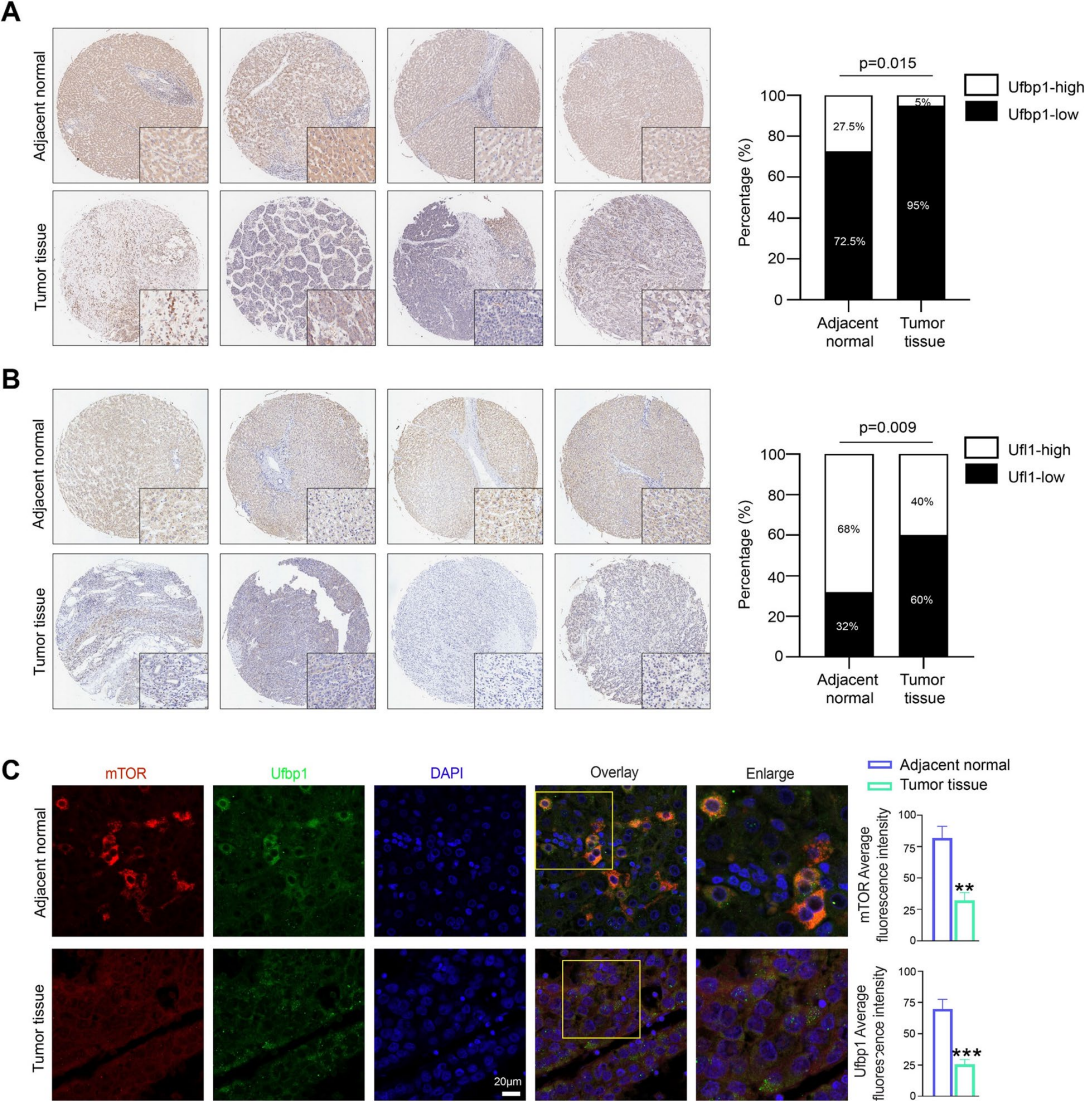
Expression of Ufl1 and Ufbp1 in Human HCC tissues. **A**, **B** Representative sections of immunostaining with Ufl1 and Ufbp1 in HCC tissues (*n* = 75) and paired adjacent normal tissues (*n* = 75) in a tissue array. **C** Immunofluorescence staining showing colocalized Ufbp1 and mTOR proteins and their intensity in human HCC tissues (*n* = 6) and paired adjacent normal tissues (*n* = 6). Quantitative data are shown in the right panel. ** *p* < 0.01; *** *p* < 0.001

Human HCC is a highly metastatic cancer with an extremely poor prognosis. Compared with adjacent normal tissues, Ufbp1 and Ufl1 expression was found to be decreased in a significant number of human HCC tissues by microarray analysis (Fig. 6A-B). Immunofluorescence analysis revealed the colocalization of Ufbp1 and mTOR in both normal and HCC liver tissues (Fig. 6C). Similar to the above findings in mice, the protein levels of Ufbp1 and mTOR were found to be lower in HCC samples than in normal liver tissue samples (Fig. 6C). Given that mTOR activation plays an important role in liver pathology and tumorigenesis, it appears that Ufl1 and Ufbp1 may serve as inhibitors of mTOR signaling to protect the liver from injury and carcinogenesis.

### Loss of hepatic *Ufl1* increases the susceptibility to DEN-induced HCC via the mTOR signaling pathway

**Fig. 7:**
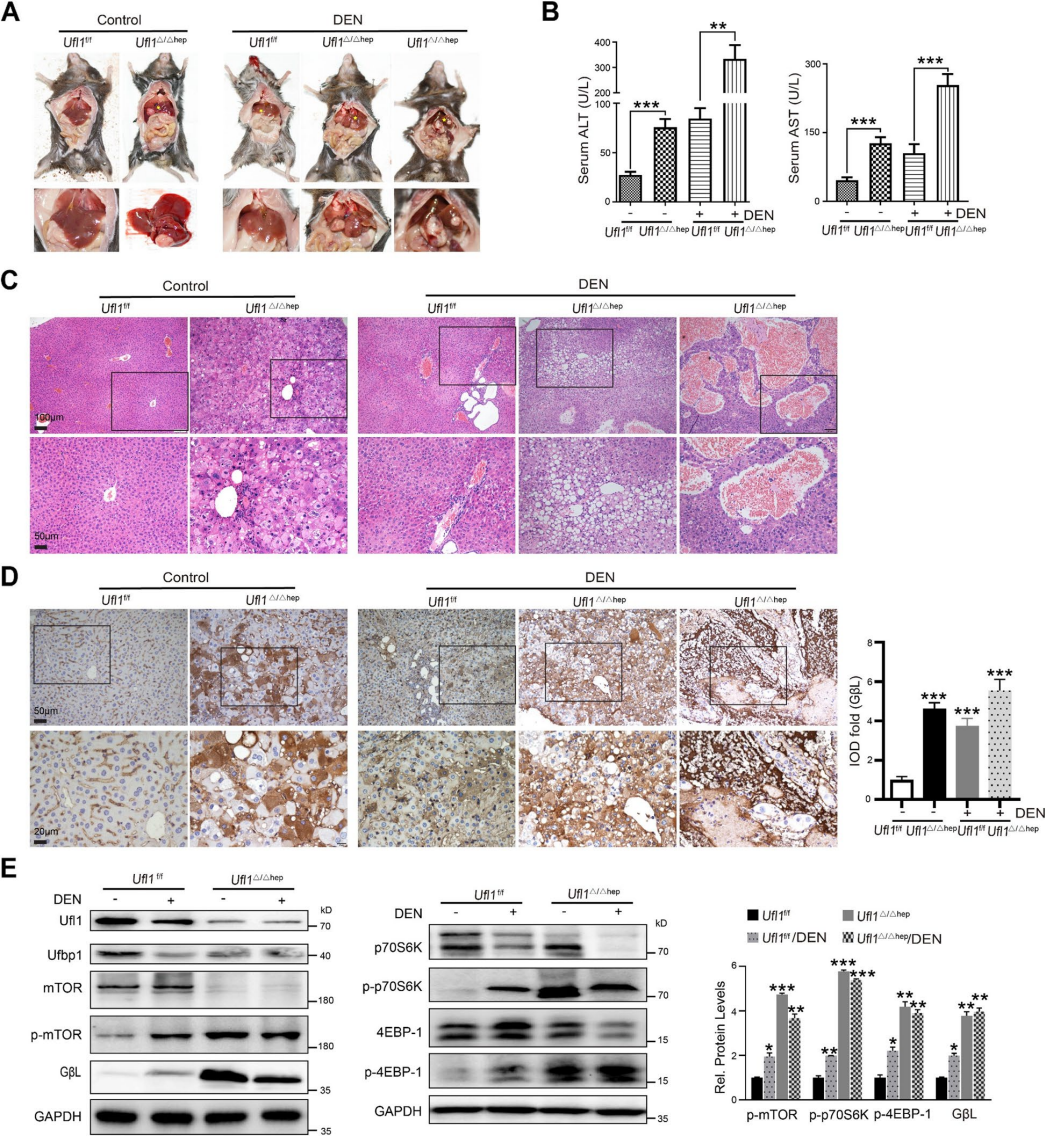
Hepatic *Ufl1* deficiency promotes liver tumor formation in DEN-induced hepatocarcinogenesis. **A** Anatomic illustration of liver tumors in 8-month-old *Ufl1*^Δ/Δhep^ and its control mice after prolonged exposure to DEN. **B** Assessment of liver injury in *Ufl1*^Δ/Δhep^ and its control mice with or without DEN induction through testing of serum ALT and AST levels (*n* = 5 mice/group). **C**, **D** Histochemical analysis and GβL immunohistochemical staining of liver tissues extracted from 8-month-old *Ufl1*^Δ/Δhep^ and control mice (*n* = 5 mice/group). **E** Western blot analysis of the key molecules involved in mTOR signaling using the samples extracted from 8-month-old *Ufl1*.^Δ/Δhep^ and control mice. All western blots were independently repeated at least three times with consistent results. Quantitative data are shown in the right panel. * *p* < 0.05; ** *p* < 0.01; *** *p* < 0.001

To further study the relationship between *Ufl1* loss and liver carcinogenesis, we treated *Ufl1*^f/f^ and *Ufl1*^Δ/Δhep^ mice with a low concentration of DEN (12 mg/kg) via injection at 6 weeks and 14 weeks of age and fed all groups a normal diet (Supplementary Fig. S6A). At 8 months of age, the liver tissue of almost all DEN-treated *Ufl1*^f/f^ mice remained smooth, soft, and bright in color, while nearly all DEN-treated *Ufl1*^Δ/Δhep^ mice suffered from liver cancer nodules and hepatocyte edema, showing very little healthy hepatic tissue. Notably, more than half of the untreated *Ufl1*^Δ/Δhep^ mice also displayed white cancer nodules and a greater degree of liver surface roughness compared with *Ufl1*^f/f^ mice treated with DEN (Fig. 7A and Supplementary Fig. S6B). The concentrations of serum ALT and AST also increased dramatically after DEN induction, as well as following *Ufl1* loss, implying severe hepatic impairment (Fig. 7B). H&E staining of liver sections from mice revealed extensive hepatocyte ballooning and cytoplasmic vacuolization, inflammatory cell infiltration, areas of necrosis, and cancer nodules after DEN induction or *Ufl1* loss (Fig. 7C).

Importantly, the liver sections from DEN-treated *Ufl1*^Δ/Δhep^ mice were characterized by a higher degree of inflammatory cell infiltration and necrosis spots surrounded by hyperplastic and hypervascular liver tissue when compared with DEN-treated *Ufl1*^f/f^ mice (Fig. 7C). Interestingly, immunoblotting of 8-month-old *Ufl1*^Δ/Δhep^ mice revealed the levels of p-mTOR, GβL, p-p70S6, and p-4EBP-1 to be markedly increased compared with age- and sex-matched KO control mice, regardless of DEN treatment, while the levels of mTOR, p70S6, 4EBP-1, Ufl1, and Ufbp1 decreased as the disease progressed (Fig. 7E). GβL staining was markedly stronger in the KO groups than in the WT groups with or without DEN treatment (Fig. 7D). These observations indicate that loss of hepatic *Ufl1* increases the susceptibility to DEN-induced HCC, at least in part via the mTOR pathway. Meanwhile, the classic phenotype of liver cancer was not observed in *Ubpl1*^Δ/Δhep^ mice with the same treatment. However, substantial pathological damage of liver tissues in mice was shown by H&E staining (Supplementary Fig. S6C).

### The mTOR/GβL complex is a downstream target of Ufl1/Ufbp1

**Fig. 8:**
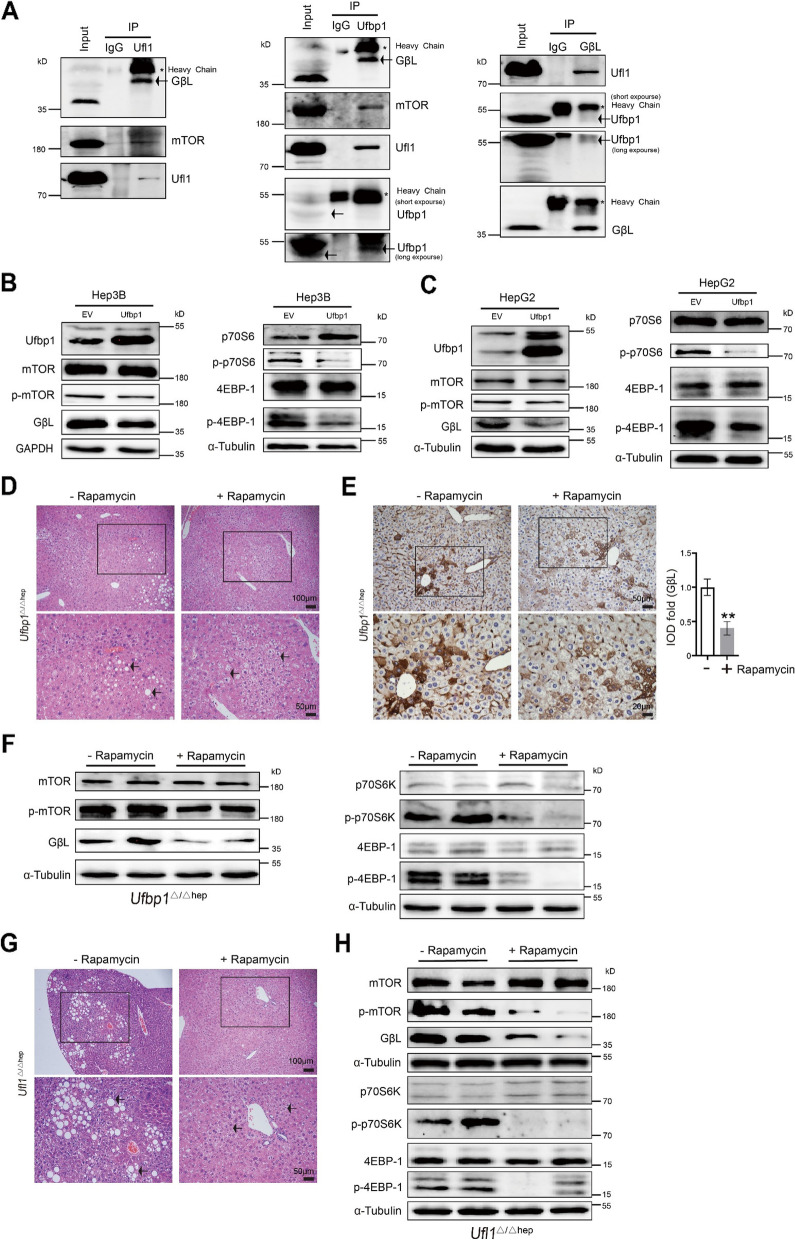
The mTOR/GβL complex is a downstream target of the Ufl1/Ufbp1 complex. **A** IP analysis showing the interaction of Ufl1 or Ufbp1 with the mTOR/GβL complex. Tissue lysates derived from 2-month-old wild-type mice were immunoprecipitated using anti-Ufl1 or Ufbp1 antibody and immunoblotted with antibodies against GβL, mTOR, Ufl1 and Ufbp1. IP assays showing the interaction of GβL with the Ufl1/Ufbp1 complex. IgG served as a negative control in IP assays. **B**, **C** Western blot analysis showing that Ufbp1 overexpression deactivates mTOR signaling in human HCC Hep3B and HepG2 cells. **D** Histochemical analysis of liver tissues extracted from 7-month-old *Ufbp1*^Δ/Δhep^ mice treated with or without rapamycin. Black arrows indicate lipid-laden hepatocytes. **E** Immunohistochemical staining for GβL in 7-month-old *Ufbp1*^Δ/Δhep^ and control mice treated with or without rapamycin. Quantitative data are shown in the right panel (*n* = 5 mice/group). **F** Western blot analysis of the key molecules involved in mTOR signaling using the samples extracted from 7-month-old *Ufbp1*^Δ/Δhep^ and control mice treated with or without rapamycin (*n* = 5 mice/group). **G** Histochemical analysis of liver tissues extracted from 7-month-old *Ufl1*^Δ/Δhep^ mice treated with or without rapamycin. Black arrows indicate lipid-laden hepatocytes. **H** Western blot analysis of the key molecules involved in the mTOR signaling using the samples extracted from 7-month-old *Ufl1*.^Δ/Δhep^ and control mice treated with or without rapamycin. All western blots were independently repeated at least three times with consistent results. ** *p* < 0.01

An important question was whether the mTOR/GβL complex was a bona fide Ufl1/Ufbp1 substrate. To address this question, IP analysis confirmed that Ufl1 and Ufbp1 were capable of binding to mTOR/GβL and that endogenous Ufl1/Ufbp1 and GβL interacted in the liver tissue of normal mice (Fig. 8A). At the same time, Ufbp1 overexpression attenuated the levels of GβL and p-mTOR, as well as the expression of downstream p-p70S6 and p-4EBP-1 in human HCC Hep3B and HepG2 cells (Fig. 8B-C). Taken together, these results suggest that the mTOR/GβL complex is indeed a bona fide substrate of the Ufl1/Ufbp1 complex.

The notion that mTOR is a downstream target of Ufl1 or Ufbp1 was also supported when we took advantage of rapamycin, a pharmacological inhibitor of mTOR, to examine whether liver damage could be slowed in *Ufbp1*^Δ/Δhep^ and *Ufl1*^Δ/Δhep^ mice through blocking mTOR activation. In brief, 6-month-old *Ufbp1*^Δ/Δhep^ mice were intraperitoneally (*i.p.*) treated with rapamycin (2 mg/kg) every other day for 2 weeks and then given 2 weeks for drug absorption before being sacrificed (Supplementary Fig. S6D). Compared with untreated *Ufbp1*^Δ/Δhep^ mice, the liver samples from rapamycin administered *Ufbp1*^Δ/Δhep^ mice exhibited attenuated hepatic steatosis, as well as decreased cytoplasmic vacuolation, inflammatory cell infiltration, and phosphorylation levels of mTOR, p70S6, 4EBP-1, and GβL (Fig. 8D and F). Immunohistochemistry staining confirmed that administration of rapamycin downregulated GβL expression in the liver tissues of *Ufbp1*^Δ/Δhep^ mice (Fig. 8E). *Ufl1*^Δ/Δhep^ mice exhibited similar results after rapamycin treatment (Fig. 8G and H). These results indicate that the Ufl1/Ufbp1 complex acts upstream of mTOR and directly regulates its activation.

## Discussion

Liver fibrosis is a pathological process characterized by excessive ECM deposition secondary to chronic hepatic injury and HSC activation. Although aimed at organ homeostasis, fibrosis can cause structural disruptions to the liver that lead to dysfunction, increased HCC likelihood, and eventually death [1]. Given the ER’s interplay with other cellular organelles and critical importance to hepatocyte metabolism, a significant emphasis has been placed on understanding the role of ER stress in liver disease, subsequently revealing its contribution to apoptosis, steatosis, inflammatory responses, and fibrosis [8]. Previous studies have reported that UFMylation is upregulated following ER stress and that the Ufm1 E3 ligase components Ufl1 and Ufbp1 play an essential role in preventing ER stress-induced apoptosis in secretory cells [9, 10]. Ufm1 members have also been shown to be downregulated in alcoholic hepatitis and cirrhosis biopsies [33], and Ufl1 and Ufbp1, particularly, are suggested to act as tumor suppressors in certain cancers [34]. Understanding the importance of the Ufm1 conjugate system, including its E3 ligase components and signaling effectors, may be important to the development of effective treatment strategies for liver disease in the future. In this study, the functional roles of Ufl1 and Ufbp1 in the liver were investigating in hepatocyte specific *Ufl1*^Δ/Δhep^ and *Ufbp1*^Δ/Δhep^ mice and revealed that deletion of *Ufl1* or *Ufbp1* leads to liver pathological changes through mTOR pathway activation.

By 2 months of age, the livers of *Ufl1*^Δ/Δhep^ and *Ufbp1*^Δ/Δhep^ mice exhibited local lesions, early signs of fibrosis, and mild hepatocyte steatosis with increased mitochondrial and nuclear abnormalities, as well as increased rates of apoptosis. Compared to KO control mice, the liver levels of α-SMA and collagen I and serum levels of ALT and AST were also significantly higher in KO mice at 6 months old, suggesting extensive liver fibrosis and injury. Administration of an HFD (commonly used to induce NAFLD) [35], further amplified hepatic injury in *Ufl1*^Δ/Δhep^ and *Ufbp1*^Δ/Δhep^ mice, leading to markedly heavier livers with multiple lesions and signs of severe steatosis with early cirrhosis, as well as an increase in proliferated fatty hepatocytes and fibroblasts and fibrosis. Most notably, more than half of the KO mice suffered from HCC and exhibited deformed liver architecture at 14–16 months old. Using the HFD as a model, it has been shown that a high-fat diet can lead to obesity and insulin resistance and increase the risk of developing type 2 diabetes. A limitation of the present study is the lack of detailed data on the function of Ufl1 or Ufbp1 in obesity and diabetes, which is critical to fully understand the novel role of these proteins in human disease.

Growing evidence has implicated aberrant mTOR activation in liver fibrosis and pathogenesis [36, 37]. We found that the Cre-mediated deletion of *Ufbp1* in MEFs led to mTOR signaling pathway activation and increased p-mTOR expression and cytoplasmic localization. In *Ufl1*^Δ/Δhep^ and *Ufbp1*^Δ/Δhep^ mice, mTOR signaling was also significantly increased compared to KO control mice and was even further increased following HFD induction. It is widely reported that HFD administration results in ER stress and UPR activation [38, 39], and there is a positive modulatory relationship between mTOR and ER stress [40, 41]. Although the interplay between HFD induction and mTOR activation in our study warrants further investigation, the two may be linked by ER stress in the absence of Ufm1 E3 ligase components. Upregulation of the mTOR pathway is seen in most cancers and, in the case of HCC, associated with earlier recurrence and poor prognosis [42, 43]. In 14-month-old mice exhibiting liver carcinogenesis, the levels of p-mTOR and GβL increased sharply following the hepatic deletion of *Ufl1* or *Ufbp1*. In addition to p-mTOR and GβL, p-p70S6 and p-4EBP-1 were also upregulated in the livers of 8-month-old *Ufl1*^Δ/Δhep^ mice relative to KO controls, regardless of DEN treatment, strongly implicating a tumor-suppressing role for Ufl1 and Ufbp1.

Collagen I was the most downregulated protein upon Ufbp1 depletion, and decreased collagen I levels were confirmed in MEFs from E14.5d *Ufbp1*^f/f^: Rosa26Cre-ERT2 mouse embryos treated with 4-OHT vs EtOH (Supplementary Fig. S7). However, this result contrasts with the in vivo observations showing increased collagen I levels in *Ufbp1*^Δ/Δhep^ mice compared with control mice. This discrepancy may be due to the cell context (MEFs vs. hepatocytes). Since mTOR upregulation upon Ufbp1 depletion was consistent in both in vitro and in vivo, we focused more on this pathway. After obtaining evidence that the mTOR/GβL complex is a bona fide substrate of the Ufl1/Ufbp1 complex, we found that rapamycin inhibition of mTOR attenuated liver damage, as well as the phosphorylation of mTOR signaling pathway members in *Ufbp1*^Δ/Δhep^ mice. Besides genetic level regulation, mTOR signaling pathway activation is influenced by post-translational modifications. mTOR inhibitory protein degradation through ubiquitination, for example, can lead to mTOR activation, whereas mTOR activating protein degradation can cause mTOR inactivation [44]. Protein modifications are, therefore, essential to maintaining proper signaling under homeostatic conditions but can have serious physiological consequences when inappropriately repressed or activated [45]. Together, our findings suggest that Ufl1 and Ufbp1 serve a protective role in the liver by regulating the activation of the mTOR signaling pathway, likely through the direct binding and UFMylation of mTOR/GβL.

Structurally, mTOR is divided into two distinct complexes, mTORC1 and mTORC2. Raptor and GβL, together with mTOR, represent the core components of mTORC1, with GβL playing a notable role in mTOR kinase activity through its binding to mTOR’s catalytic domain. mTORC2 also involves mTOR and GβL but includes Rictor rather than Raptor. Using 2-month-old *Ufbp1*^Δ/Δhep^ and *Ufl1*^Δ/Δhep^ mice, we found that while the levels of Rictor protein in livers were unaffected by *Ufbp1* or *Ufl1* loss, the levels of Raptor protein decreased in the absence of *Ufl1* but not *Ufbp1*. Discordantly, however, the expression of Raptor protein was found to be upregulated in *Ufbp1* KO embryos in most instances, while Rictor expression was again unaffected. As this discrepancy and the overall minimal effect that *Ufl1* or *Ufbp1* loss had on Raptor and Rictor, we subsequently decided to focus our attention on this investigation instead on mTOR, GβL, and downstream signaling molecules, like p70S6K and 4EBP-1, whose direct phosphorylation by mTOR promotes protein synthesis. In future studies, delineating the involvement of mTORC1 and mTORC2 and their regulation by Ufm1 E3 ligase components will be critical to expanding the therapeutic potential of these findings.

**Fig. 9:**
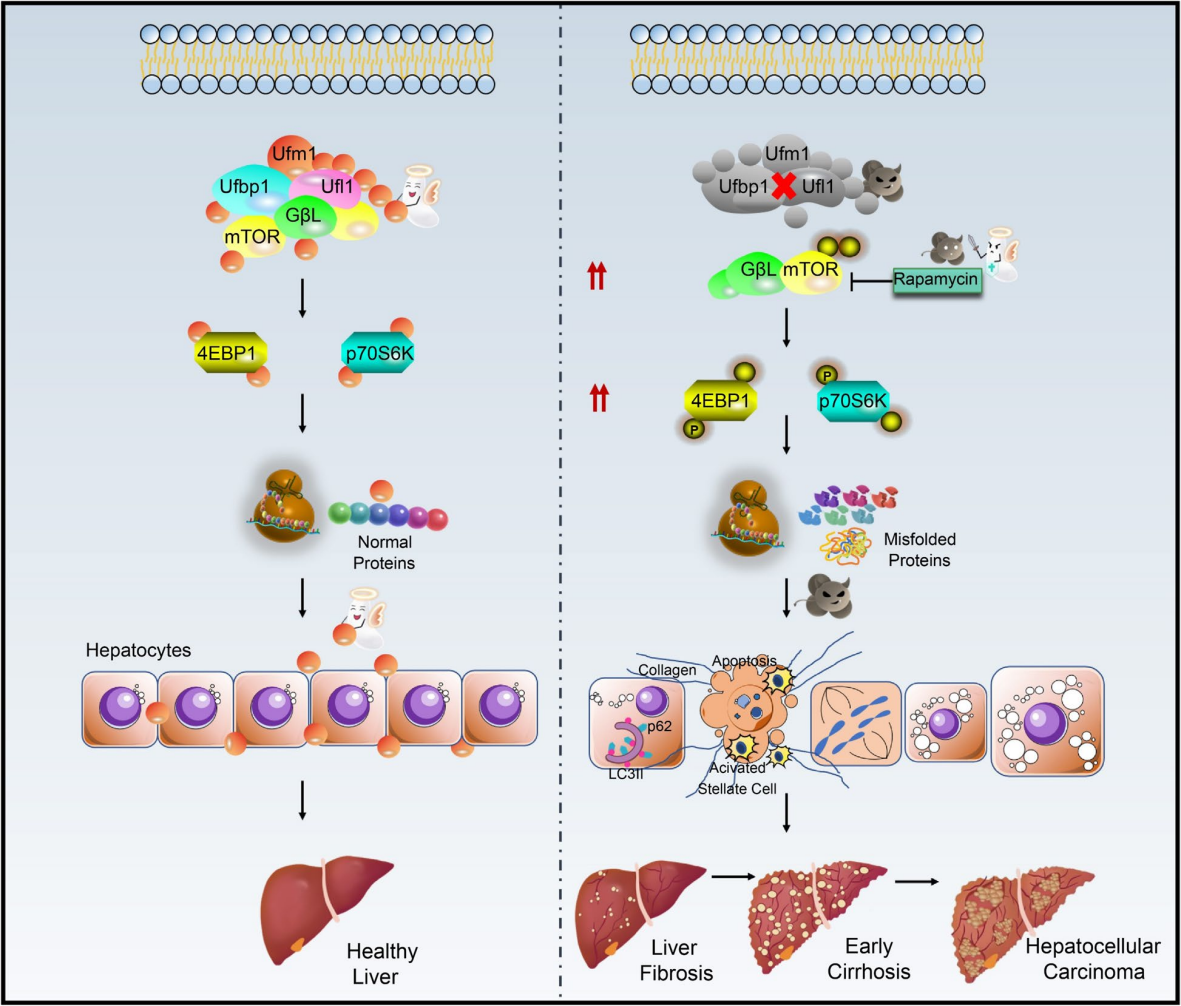
Ufl1 and Ufbp1-dependent-UFMylation acts in a protective role in maintaining liver homeostasis. Aberrant upregulation of p-mTOR and GβL results from Ufl1 and Ufbp1 deficiency in the liver, which also leads strongly to p70S6K and 4EBP1 phosphorylation. The chronic and abnormal activation of mTOR signaling pathway results in the accumulation of misfolded proteins, which induces liver damage. Hepatocyte steatosis and fibrosis occur in *Ufl1*^Δ/Δhep^ and *Ufbp1*^Δ/Δhep^ mice at the age of 6–8 weeks initially. Ballooning and steatohepatitis develop at 8-month-old, and more than a half of *Ufl1*^Δ/Δhep^ mice develop liver cancer at about 14-month-old. The mTOR inhibitor rapamycin attenuates the extent of hepatic steatosis partially through inhibiting the mTOR signaling pathway

While the potential mechanism by which mTOR activation leads to liver injury is not investigated, it is possible that excessive mTOR signaling provokes ER stress and UPR dysregulation [46], leading to apoptosis, fibrosis, and the other liver pathologies seen [8]. Hepatic loss of Ufl1 and Ufbp1 impairs mTOR function, causing HSC activation, fat accumulation overload and apoptosis in hepatocytes. The balance of liver homeostasis is disrupted directly by Ufl1/Ufbp1 deficiency. In summary, Ufl1/Ufbp1 provides a potential druggable target for treating liver disease in humans (Fig. 9). Park et al*.* reported that Sestrin-2 KO mice triggered ER stress-associated severe liver injuries, such as steatohepatitis and fibrosis, by inducing excessive ER stress-associated cell death [47]. Fibrosis resulting from overactive PI3K/AKT/mTOR signaling promotes collagen production and the activation of HSCs. Myofibroblasts and fibrosis may then subsequently influence liver pathologies, including the development of HCC through modulation of the tumor microenvironment by fibronectin-mediated cell growth stimulation or cytokine and growth factor release [6, 48, 49]. Needless to say, the molecular influence of mTOR in the liver is expansive and its regulation by Ufm1 E3 ligase will require future study.

Autophagy allows the body’s cells to break down fat molecules and use them for energy production [50]. This can be particularly important during periods of nutrient deprivation, such as fasting or calorie restriction, when the body needs to generate energy from stored fat. Other conditions, such as obesity and insulin resistance, can impair autophagy and promote fat accumulation in the body. It's worth noting that autophagy is a complex process involving many different mechanisms and factors, and the relationship between autophagy and fat levels in the body is not yet fully understood. In the present study, increased autophagy and higher fat levels were observed in mouse livers deficient in Ufl1 or Ufbp1. These findings suggest that loss of Ufl1/Ufbp1 in hepatocytes promotes liver steatosis as a separate event from mTOR-dependent autophagy, although investigation of the underlying mechanism is warranted.

The reduction of mTOR hyperactivation in *Ufbp1*^Δ/Δhep^ and *Ufl1*^Δ/Δhep^ mice after 2 weeks of rapamycin treatment is expected, but the concomitant reduction of pathological changes in such a strong fibrotic phenotype is surprising and may involve other effects of rapamycin on different cells, such as HSCs, the main cells inducing fibrotic changes. As shown in Supplementary Fig. S8, rapamycin treatment dramatically reduced α-SMA immunointensity in these KO mice, suggesting a possible role of HSCs in the induction of fibrotic changes. The reduction of lipid vacuoles with rapamycin may not be due to direct regulation of the Ufl1/Ufbp1-mTOR signaling axis in hepatocytes, which needs to be investigated and analyzed in the future study.

## Conclusions

In conclusion, hepatocyte-specific deletion of *Ufl1* or *Ufbp1* in mice increases the susceptibility to HFD-induced fatty liver and DEN-induced HCC. The Ufl1/Ufbp1 complex functions as a novel upstream repressor of mTOR activation, and thereby serving as a defender of liver homeostasis and protecting against liver damage. Although these findings highlight the importance of Ufm1 E3 ligase components, alternative mechanisms cannot be excluded and therefore warrant further investigations. Nevertheless, this study sheds new light on theoretical and clinical researchers focusing on liver diseases by expanding our understanding of Ufm1 E3 ligase.

## Supplementary Information

**Additional file 1: Table S1.** Commercial primary antibodies used in the study. **Table S2.** List of primers used for qRT-PCR. **Table S3.** Summary of the top 15 upregulated and downregulated DE proteins identified from iTRAQ assays (4-OHT vs EtOH). **Supplementary Fig. S1. **(A) KO efficiency determined by western blot. (B) Ratio of liver weight to body weight in 2-month-old *Ufbp1*^Δ/Δhep^ mice. ** *p*<0.01. **Supplementary Fig. S2. **(A) mRNA expression analysis of Collagen I after Ufl1 or Ufbp1 loss at the age of 6 months old. (B) Timeline of HFD treatment in *Ufl1*^Δ/Δhep^ or *Ufbp1*^Δ/Δhep^ mice, and ratios of the liver to body weight in *Ufl1*^Δ/Δhep^, *Ufbp1*^Δ/Δhep^, and their control mice after 12 weeks of HFD feeding. *** *p*<0.001. **Supplementary Fig. S3. **(A) Cell viability of 4-OHT-treated and EtOH-treated groups assessed by crystal violet staining on day 6. (B) GO term enrichment for Biological Process, Cellular Component, and Molecular Function. (C) Genotypes of mouse embryo. (D) Raptor and Rictor protein levels in E11.5d embryos from *Ufbp1* KO mice. ** *p*<0.01. **Supplementary Fig. S4.** *Ufl1* or *Ufbp1* deficiency induces autophagy. (A) Raptor and Rictor protein levels in 2-month-old *Ufl1*^Δ/Δhep^, *Ufbp1*^Δ/Δhep^, and control mice. (B) LC3B and p62 proteins in 8-month-old *Ufl1*^Δ/Δhep^, *Ufbp1*^Δ/Δhep^, and control mice. (C) Immunohistochemical staining of LC3B in 8-month-old *Ufbp1*^Δ/Δhep^ and control mice. Quantitative data are shown in the right panel (n=5 mice/group). (D) Immunofluorescence of LC3B in *Ufbp1*^-/-^ MEFs. Quantitative data are shown in the right panel (n=5 mice/group). * *p*<0.05; ** *p*<0.01; *** *p*<0.001. **Supplementary Fig. S5. **(A) Tumor incidence of *Ufl1*^Δ/Δhep^, *Ufbp1*^Δ/Δhep^, and their control mice at the age of 14 months old. (B) AFP gene expression in *Ufbp1*^Δ/Δhep^ mouse livers at the age of 2 months and 14 months old. Quantitative data are shown in the right panel. *** *p*<0.001. **Supplementary Fig. S6. **(A) Timeline of experimental procedures used to generate DEN-induced hepatocarcinogenesis. (B) Tumor incidence of *Ufl1*^Δ/Δhep^ and its control mice with or without DEN induction. (C) Histopathological analysis of liver tissues extracted from 8-month-old *Ufbp1*^Δ/Δhep^ and control mice with or without DEN induction. (D) Timeline of rapamycin treatment in *Ufbp1*^Δ/Δhep^ and *Ufl1*^Δ/Δhep^ mice. **Supplementary Fig. S7. **Comparison of collagen I protein levels in MEFs from E14.5d Ufbp1^f/f^: Rosa26Cre-ERT2 mouse embryos treated with 4-OHT vs. EtOH. **Supplementary Fig. S8. **Immunostaining of α-SMA in liver tissues extracted from 7-month-old *Ufbp1*^Δ/Δhep^ or *Ufl1*^Δ/Δhep^ mice treated with or without rapamycin. Quantitative data are shown in the right panel (n=5 mice/group). ** *p*<0.01.

## Data Availability

All data generated or analyzed during this study are included in this published article [and its supplementary information files].

## Abbreviations

ALT: Alanine aminotransferase
AST: Aspartate aminotransferase
DE: Differentially expressed
DEN: Diethylnitrosamine
ECM: Extracellular matrix
ER: Endoplasmic reticulum
EtOH: Ethyl alcohol
HCC: Hepatocellular carcinoma
HFD: High-fat diet
HSCs: Hepatic satellite cells
MEFs: Mouse embryonic fibroblasts
mTOR: Mammalian target of rapamycin
NAFLD: Non-alcoholic fatty liver disease
TG: Triglyceride
Ufbp1: Ufm1-binding protein 1
Ufl1: Ufm1-specific ligase 1
Ufm1: Ubiquitin-fold modifier 1
