# Supplementary material for: Loss of Ufl1/Ufbp1 in hepatocytes promotes liver pathological damage and carcinogenesis through activating mTOR signaling
Source: J Exp Clin Cancer Res. 2023 May 3;42:110. doi: 10.1186/s13046-023-02681-6 (PMC10155312; doi:10.1186/s13046-023-02681-6)
Supplement: Supplementary file 1 — Additional file 1: Table S1. Commercial primary antibodies used in the study. Table S2. List of primers used for qRT-PCR. Table S3. Summary of the top 15 upregulated and downregulated DE proteins identified from iTRAQ assays (4-OHT vs EtOH). Supplementary Fig. S1. (A) KO efficiency determined by western blot. (B) Ratio of liver weight to body weight in 2-month-old Ufbp1Δ/Δhep mice. ** p<0.01. Supplementary Fig. S2. (A) mRNA expression analysis of Collagen I after Ufl1 or Ufbp1 loss at the age of 6 months old. (B) Timeline of HFD treatment in Ufl1Δ/Δhep or Ufbp1Δ/Δhep mice, and ratios of the liver to body weight in Ufl1Δ/Δhep, Ufbp1Δ/Δhep, and their control mice after 12 weeks of HFD feeding. *** p<0.001. Supplementary Fig. S3. (A) Cell viability of 4-OHT-treated and EtOH-treated groups assessed by crystal violet staining on day 6. (B) GO term enrichment for Biological Process, Cellular Component, and Molecular Function. (C) Genotypes of mouse embryo. (D) Raptor and Rictor protein levels in E11.5d embryos from Ufbp1 KO mice. ** p<0.01. Supplementary Fig. S4. Ufl1 or Ufbp1 deficiency induces autophagy. (A) Raptor and Rictor protein levels in 2-month-old Ufl1Δ/Δhep, Ufbp1Δ/Δhep, and control mice. (B) LC3B and p62 proteins in 8-month-old Ufl1Δ/Δhep, Ufbp1Δ/Δhep, and control mice. (C) Immunohistochemical staining of LC3B in 8-month-old Ufbp1Δ/Δhep and control mice. Quantitative data are shown in the right panel (n=5 mice/group). (D) Immunofluorescence of LC3B in Ufbp1-/- MEFs. Quantitative data are shown in the right panel (n=5 mice/group). * p<0.05; ** p<0.01; *** p<0.001. Supplementary Fig. S5. (A) Tumor incidence of Ufl1Δ/Δhep, Ufbp1Δ/Δhep, and their control mice at the age of 14 months old. (B) AFP gene expression in Ufbp1Δ/Δhep mouse livers at the age of 2 months and 14 months old. Quantitative data are shown in the right panel. *** p<0.001. Supplementary Fig. S6. (A) Timeline of experimental procedures used to generate DEN-induced hepatocarcinogenesis. (B) Tumor in [file 13046_2023_2681_MOESM1_ESM.docx]

**Loss of Ufl1/Ufbp1 in hepatocytes promotes liver pathological damage and carcinogenesis through activating mTOR signaling**

Fanghui Chen, Le Sheng, Tianci Zhou, Li Yan, Reid Loveless, Honglin Li, Yong Teng*, Yafei Cai*

***Corresponding author:** Yafei Cai: [ycai@njau.edu.cn](mailto:ycai@njau.edu.cn);

Yong Teng: [yong.teng@emory.edu](mailto:yong.teng@emory.edu)

**This PDF file includes:**

**Supplementary Tables and Figures**

**Supplementary Tables and Table Captions**

**Table S1. Commercial primary antibodies used in the study**

| **Antibodies** | **Brand** | **Catalogue Number** |
| --- | --- | --- |
| Ufl1 | Abcam | ab226216 |
| Ufbp1 | Sigma-Aldrich | HPA013373 |
| Ufbp1 | Proteintech | 21445–1-AP |
| p-mTOR (Ser2448) (D9C2) | Cell Signaling | #5536 |
| mTOR (7C10) | Cell Signaling | #2983 |
| mTOR | Abcam | Ab32028 |
| mTOR | Proteintech | 66888-1-Ig |
| Raptor (24C12) | Cell Signaling | #2280 |
| Rictor (53A2) | Cell Signaling | #2114 |
| GβL (86B8) | Cell Signaling | #3274 |
| GβL | Santa Cruz | Sc-514982 |
| P70 S6 Kinase | Cell Signaling | #9202 |
| Phospho-p70 S6 Kinase (Thr389) | Cell Signaling | #9205 |
| 4E-BP1 (53H11) | Cell Signaling | #9644 |
| Phospho-4E-BP1 (Thr37/46) | Cell Signaling | #2855 |
| AFP | Proteintech | 14550-1-AP |
| Ki67 | Invitrogen | 14-5698-82 |
| α-SMA (D4K9N) | Cell Signaling | #19245 |
| Collagen I | Boster | BA0325 |
| LC3B | Sigma-Aldrich | L7543 |
| P62 | Abcam | ab109012 |
| β-Actin (8H10D10) | Cell Signaling | #3700 |
| α-Tubulin (11H10) | Cell Signaling | #2125 |
| GAPDH (14C10) | Cell Signaling | #2118 |

**Table S2. List of primers used for qRT-PCR**

| **Gene (mouse)** | **Forward primer** | **Reverse primer** |
| --- | --- | --- |
| β-actin | GACCTCTATGCCAACACAGT | AGTACTTGCGCTCAGGAGGA |
| GAPDH | TGGATTTGGACGCATTGGTC | TTTGCACTGGTACGTGTTGAT |
| Collagen I | AAGAGGAAGACAGCACAGCC | AGCGTCAGGATCCCTCTCTT |
| AFP | CTGCTACATTTCGCTGCGTC | GTTCACTTCCTCCTCGGTGG |

**Table S3. Summary of the top 15 upregulated and downregulated DE proteins identified from iTRAQ assays (4-OHT vs EtOH)**

| **Diff-state** | **Accession** | **Name** | **Fold change** |
| --- | --- | --- | --- |
| up | sp\|Q9DCJ1\|LST8  _MOUSE | Target of rapamycin complex subunit LST8  OS=Mus musculus GN=Mlst8 PE=1 SV=1 | 7.006628884 |
| up | tr\|A2APF7\|A2APF7  _MOUSE | Z-DNA-binding protein 1  OS=Mus musculus GN=Zbp1 PE=1 SV=1 | 3.775125804 |
| up | tr\|Q3THC3\|Q3THC3  _MOUSE | Protein disulfide-isomerase  OS=Mus musculus GN=P4hb PE=2 SV=1 | 3.328227386 |
| up | tr\|A0A0A0MQF6\|A0A0A0MQF6_MOUSE | Glyceraldehyde-3-phosphate dehydrogenase  OS=Mus musculus GN=Gapdh PE=1 SV=1 | 3.294272402 |
| up | sp\|P97770\|THUM3  _MOUSE | THUMP domain-containing protein 3  OS=Mus musculus GN=Thumpd3 PE=1 SV=1 | 3.238939948 |
| up | sp\|P52480\|KPYM  _MOUSE | Pyruvate kinase PKM  OS=Mus musculus GN=Pkm PE=1 SV=4 | 3.187843737 |
| up | tr\|Q564E2\|Q564E2  _MOUSE | L-lactate dehydrogenase  OS=Mus musculus GN=Ldha PE=1 SV=1 | 2.9587303 |
| up | tr\|Q9DC41\|Q9DC41  _MOUSE | Putative uncharacterized protein  OS=Mus musculus GN=Hspa5 PE=2 SV=1 | 2.953342589 |
| up | tr\|Q8C338\|Q8C338  _MOUSE | Isocitrate dehydrogenase [NADP]  OS=Mus musculus GN=Idh1 PE=2 SV=1 | 2.777131534 |
| up | sp\|Q60930\|VDAC2  _MOUSE | Voltage-dependent anion-selective channel protein 2  OS=Mus musculus GN=Vdac2 PE=1 SV=2 | 2.723650538 |
| up | tr\|E9PV48\|E9PV48  _MOUSE | Protein Ifit3b  OS=Mus musculus GN=Ifit3b PE=1 SV=1 | 2.684441781 |
| up | tr\|Q56A15\|Q56A15  _MOUSE | Cytochrome c  OS=Mus musculus GN=Cycs PE=2 SV=1 | 2.659318121 |
| up | tr\|Q5FW97\|Q5FW97  _MOUSE | Alpha-enolase  OS=Mus musculus GN=EG433182 PE=1 SV=1 | 2.538652636 |
| up | tr\|Q3U7A2\|Q3U7A2  _MOUSE | Putative uncharacterized protein  OS=Mus musculus GN=Ifit1 PE=2 SV=1 | 2.519506518 |
| up | tr\|Q9CZU7\|Q9CZU7  _MOUSE | Putative uncharacterized protein  OS=Mus musculus GN=Lamp2 PE=2 SV=1 | 2.486745531 |
| **Diff-state** | **Accession** | **Name** | **Fold changes** |
| down | sp\|P11087\|CO1A1  _MOUSE | Collagen alpha-1(I) chain  OS=Mus musculus GN=Col1a1 PE=1 SV=4 | 0.17888825 |
| down | tr\|Q8BKY2\|Q8BKY2  _MOUSE | Putative uncharacterized protein  OS=Mus musculus GN=Col3a1 PE=2 SV=1 | 0.180296554 |
| down | tr\|Q3TUE2\|Q3TUE2  _MOUSE | Putative uncharacterized protein (Fragment)  OS=Mus musculus GN=Col1a2 PE=2 SV=1 | 0.202681792 |
| down | tr\|Q545P0\|Q545P0  _MOUSE | Sodium/potassium-transporting ATPase subunit beta OS=Mus musculus GN=Atp1b1 PE=1 SV=1 | 0.321984312 |
| down | tr\|Q78P93\|Q78P93  _MOUSE | Acid ceramidase  OS=Mus musculus GN=Asah1 PE=1 SV=1 | 0.348035575 |
| down | tr\|B1AWB9\|B1AWB9_MOUSE | Collagen alpha-1(V) chain  OS=Mus musculus GN=Col5a1 PE=1 SV=1 | 0.35475102 |
| down | tr\|Q4KML7\|Q4KML7_MOUSE | Ezrin  OS=Mus musculus GN=Ezr PE=1 SV=1 | 0.362717558 |
| down | tr\|Q8BK60\|Q8BK60  _MOUSE | Putative uncharacterized protein  OS=Mus musculus GN=Serpinb1a PE=2 SV=1 | 0.387474661 |
| down | tr\|D3Z7B5\|D3Z7B5  _MOUSE | Protein C330027C09Rik  OS=Mus musculus GN=C330027C09Rik PE=1 SV=1 | 0.393310699 |
| down | sp\|Q4VA53\|PDS5B  _MOUSE | Sister chromatid cohesion protein PDS5 homolog B OS=Mus musculus GN=Pds5b PE=1 SV=1 | 0.415988618 |
| down | sp\|Q8BH64\|EHD2  _MOUSE | EH domain-containing protein 2  OS=Mus musculus GN=Ehd2 PE=1 SV=1 | 0.42945639 |
| down | tr\|Q80YQ1\|Q80YQ1  _MOUSE | Thrombospondin 1  OS=Mus musculus GN=Thbs1 PE=1 SV=1 | 0.431974567 |
| down | tr\|Q3UCD9\|Q3UCD9  _MOUSE | Cathepsin D  OS=Mus musculus GN=Ctsd PE=1 SV=1 | 0.447069044 |
| down | tr\|E9PX70\|E9PX70  _MOUSE | Collagen alpha-1(XII) chain  OS=Mus musculus GN=Col12a1 PE=1 SV=1 | 0.454482354 |
| down | sp\|Q07797\|LG3BP  _MOUSE | Galectin-3-binding protein  OS=Mus musculus GN=Lgals3bp PE=1 SV=1 | 0.480343775 |

**Supplementary Figures and Figure Legends**


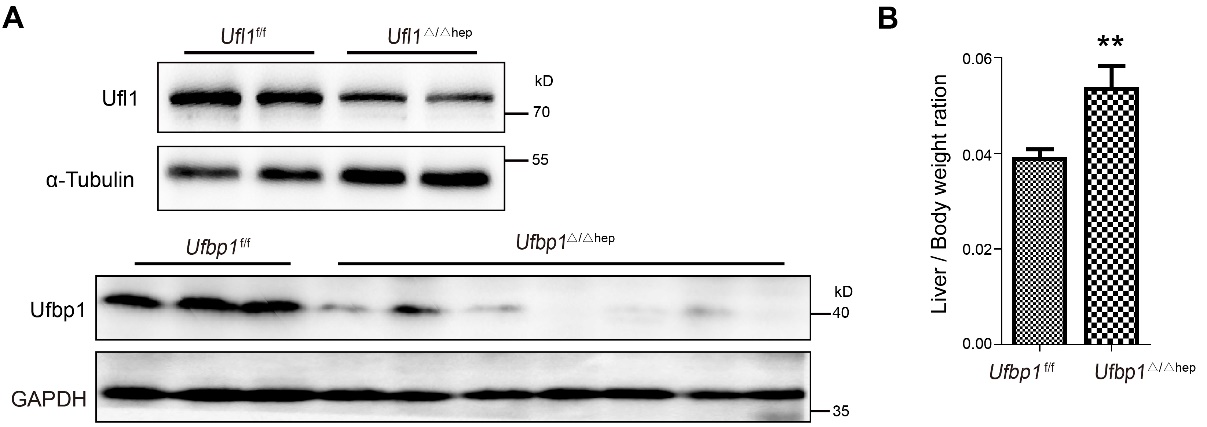


**Supplementary Fig. S1.** (A) KO efficiency determined by western blot. (B) Ratio of liver weight to body weight in 2-month-old *Ufbp1*^Δ/Δhep^ mice. ** *p*<0.01.


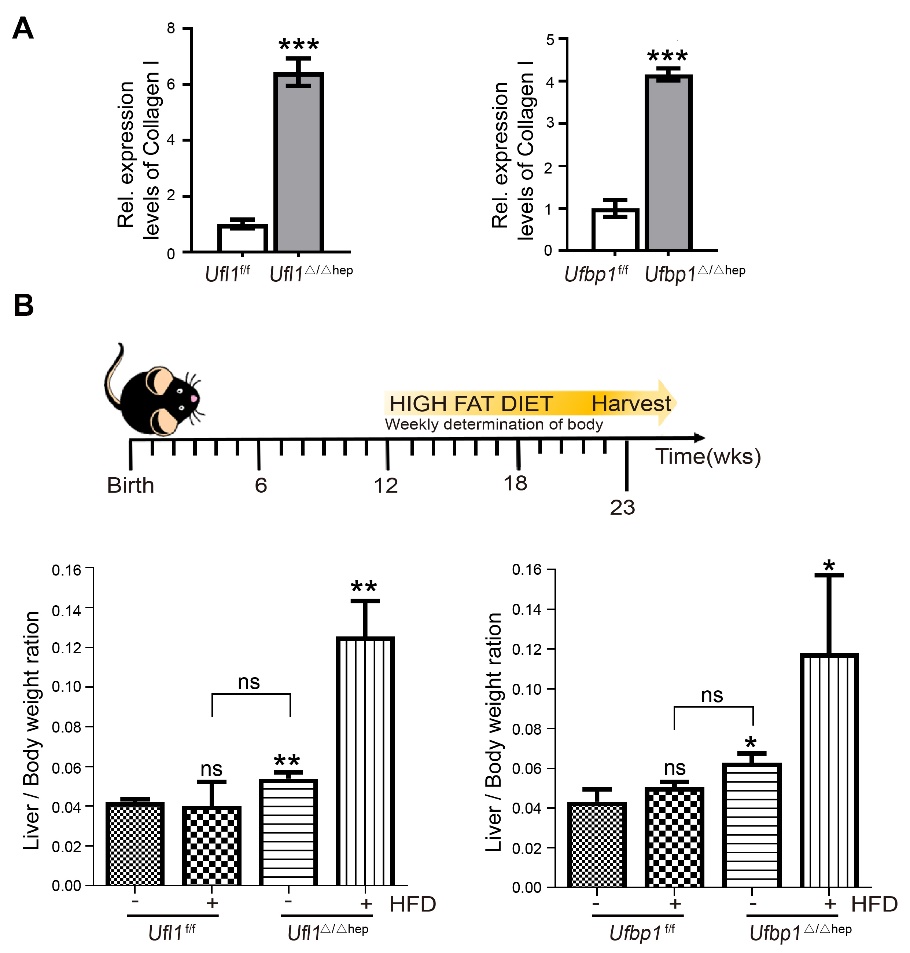


**Supplementary Fig. S2.** (A) mRNA expression analysis of Collagen I after Ufl1 or Ufbp1 loss at the age of 6 months old. (B) Timeline of HFD treatment in *Ufl1*^Δ/Δhep^ or *Ufbp1*^Δ/Δhep^ mice, and ratios of the liver to body weight in *Ufl1*^Δ/Δhep^, *Ufbp1*^Δ/Δhep^, and their control mice after 12 weeks of HFD feeding. *** *p*<0.001.


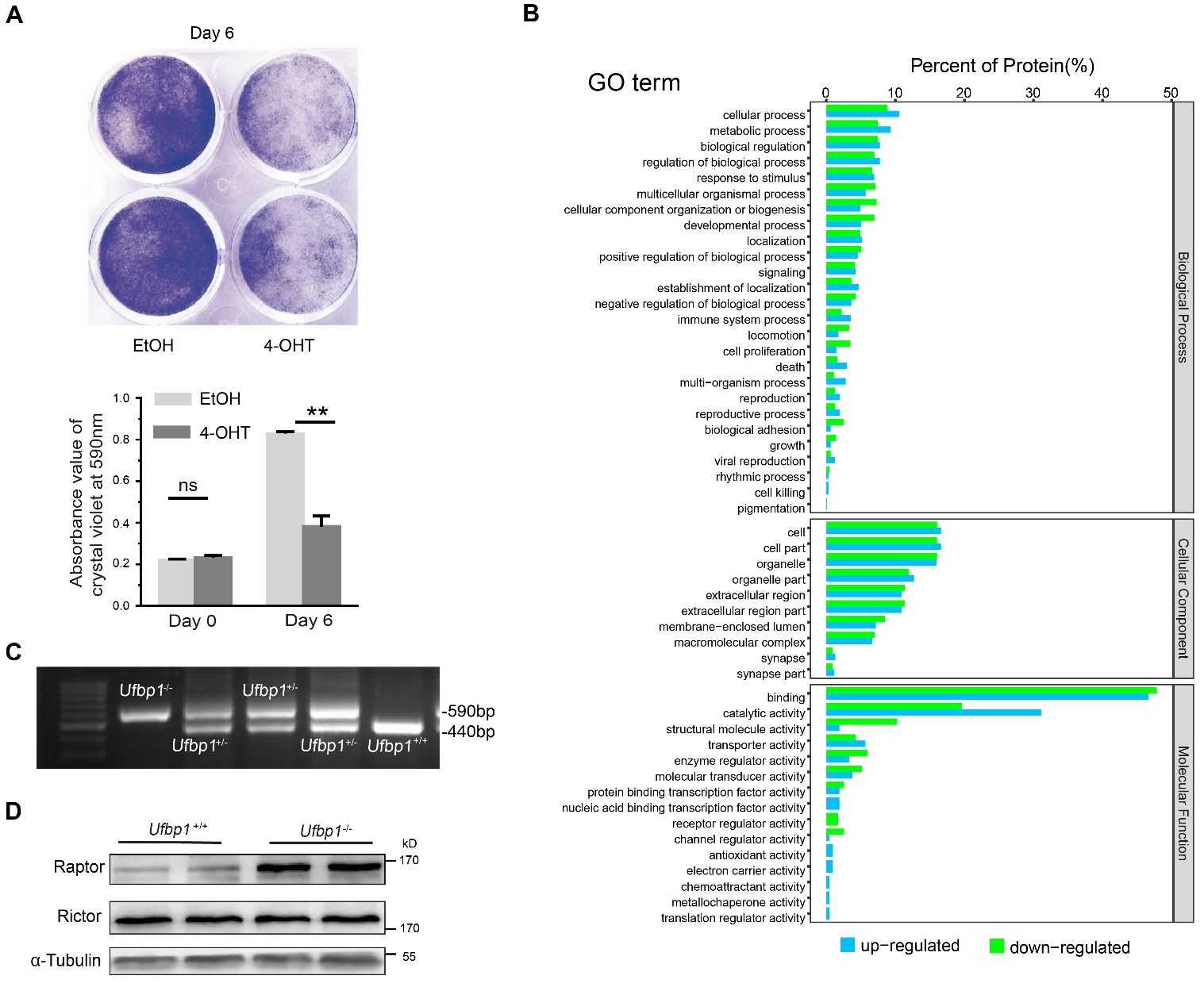


**Supplementary Fig. S3.** (A) Cell viability of 4-OHT-treated and EtOH-treated groups assessed by crystal violet staining on day 6. (B) GO term enrichment for Biological Process, Cellular Component, and Molecular Function. (C) Genotypes of mouse embryo. (D) Raptor and Rictor protein levels in E11.5d embryos from *Ufbp1* KO mice. ** *p*<0.01.


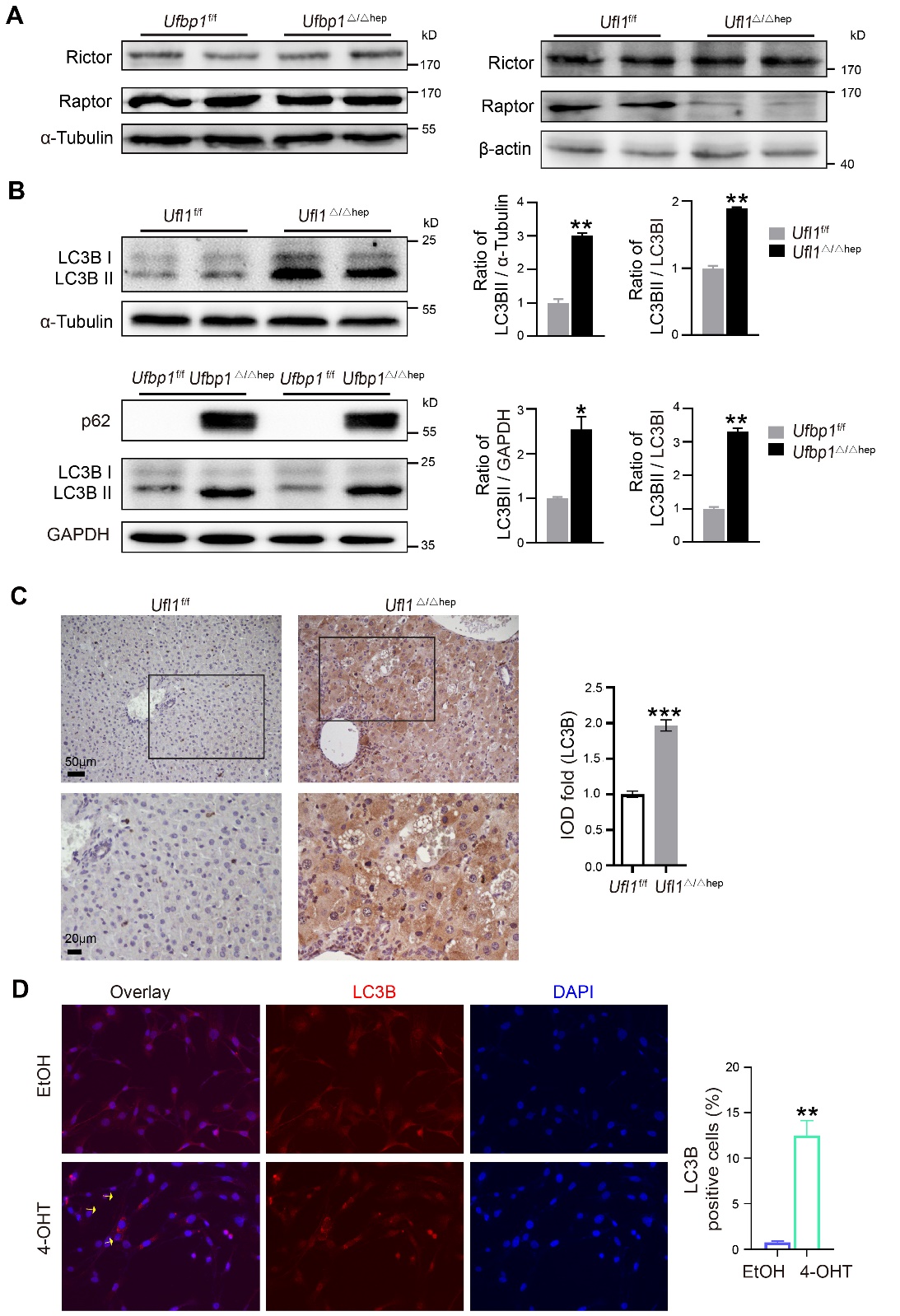


**Supplementary Fig. S4. *Ufl1* or *Ufbp1* deficiency induces autophagy.** (A) Raptor and Rictor protein levels in 2-month-old *Ufl1*^Δ/Δhep^, *Ufbp1*^Δ/Δhep^, and control mice. (B) LC3B and p62 proteins in 8-month-old *Ufl1*^Δ/Δhep^, *Ufbp1*^Δ/Δhep^, and control mice. (C) Immunohistochemical staining of LC3B in 8-month-old *Ufbp1*^Δ/Δhep^ and control mice. Quantitative data are shown in the right panel (n=5 mice/group). (D) Immunofluorescence of LC3B in *Ufbp1*^-/-^ MEFs. Quantitative data are shown in the right panel (n=5 mice/group). * *p*<0.05; ** *p*<0.01; *** *p*<0.001.


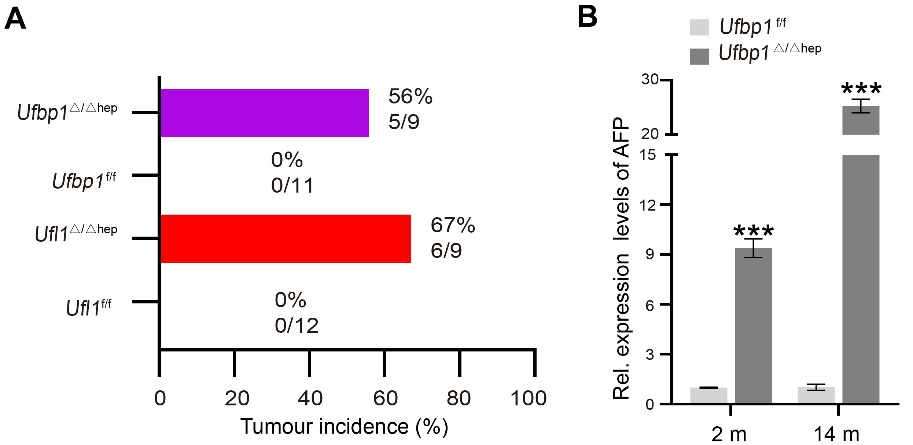


**Supplementary Fig. S5.** (A) Tumor incidence of *Ufl1*^Δ/Δhep^, *Ufbp1*^Δ/Δhep^, and their control mice at the age of 14 months old. (B) AFP gene expression in *Ufbp1*^Δ/Δhep^ mouse livers at the age of 2 months and 14 months old. Quantitative data are shown in the right panel. *** *p*<0.001.


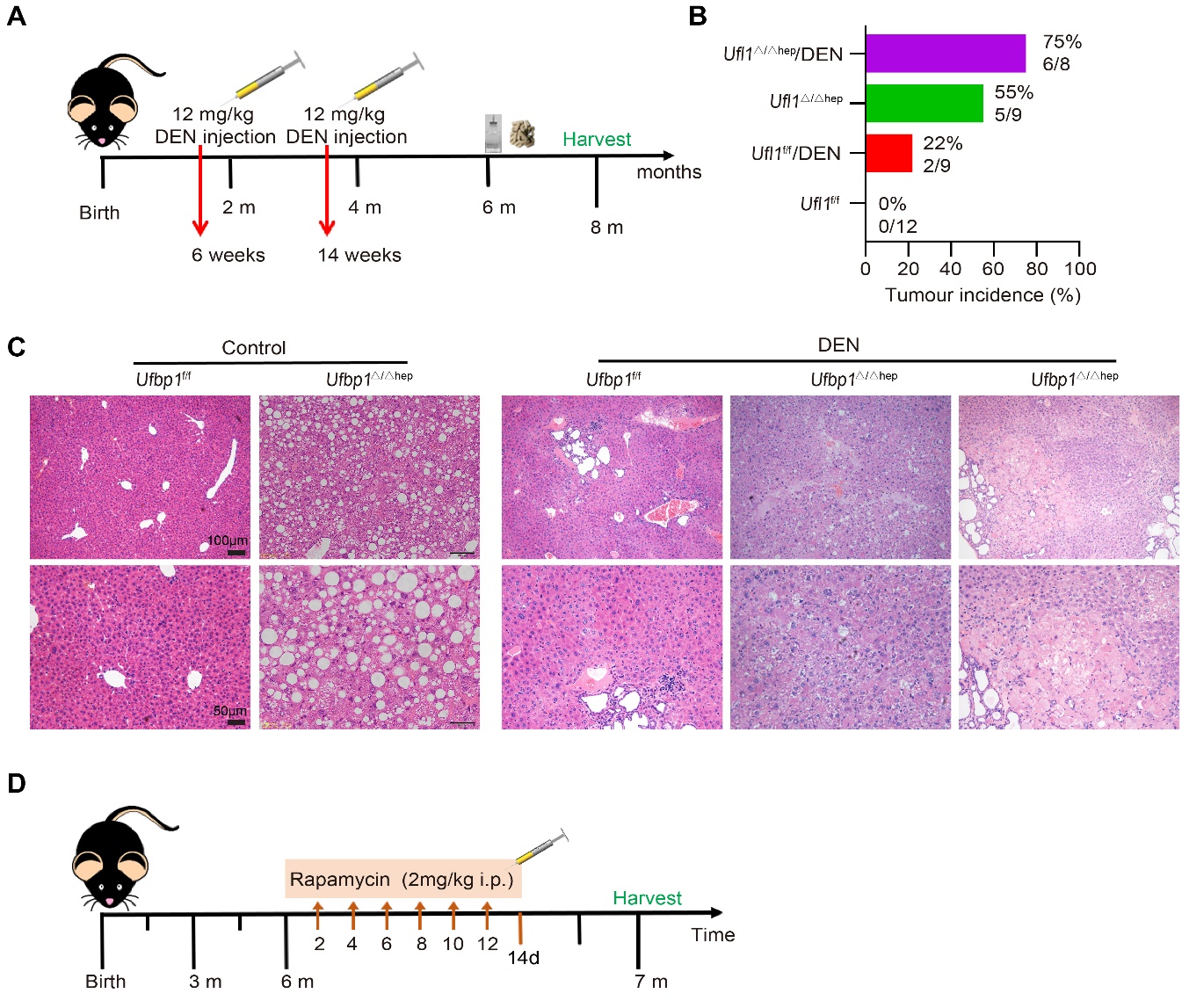


**Supplementary Fig. S6.** (A) Timeline of experimental procedures used to generate DEN-induced hepatocarcinogenesis. (B) Tumor incidence of *Ufl1*^Δ/Δhep^ and its control mice with or without DEN induction. (C) Histopathological analysis of liver tissues extracted from 8-month-old *Ufbp1*^Δ/Δhep^ and control mice with or without DEN induction. (D) Timeline of rapamycin treatment in *Ufbp1*^Δ/Δhep^ and *Ufl1*^Δ/Δhep^ mice.


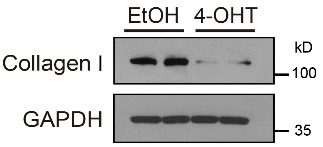


**Supplementary Fig. S7.** Comparison of collagen I protein levels in MEFs from E14.5d Ufbp1^f/f^: Rosa26Cre-ERT2 mouse embryos treated with 4-OHT vs. EtOH.


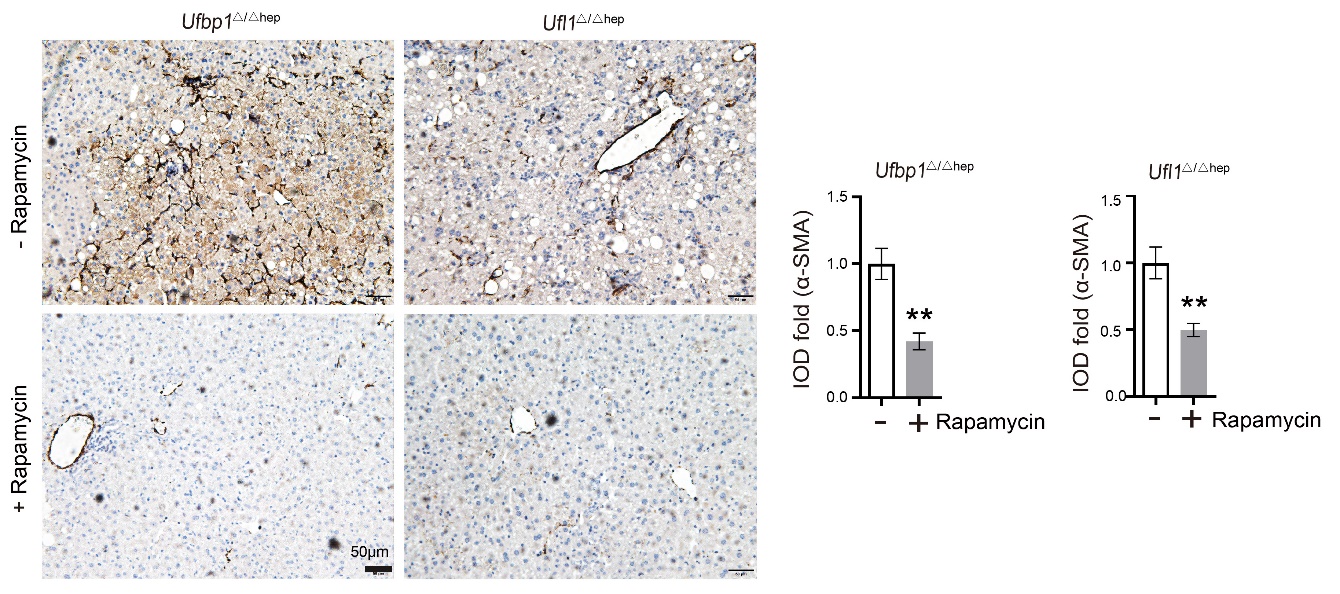


**Supplementary Fig. S8.** Immunostaining of α-SMA in liver tissues extracted from 7-month-old *Ufbp1*^Δ/Δhep^ or *Ufl1*^Δ/Δhep^ mice treated with or without rapamycin. Quantitative data are shown in the right panel (n=5 mice/group). ** *p*<0.01.
